# RNASeq highlights ATF6 pathway regulators for CHO cell engineering with different impacts of *ATF6β* and *WFS1* knockdown on fed-batch production of IgG_1_

**DOI:** 10.1038/s41598-024-64767-1

**Published:** 2024-06-19

**Authors:** Dyllan Rives, Caroline Peak, Mark A. Blenner

**Affiliations:** 1 Department of Chemical & Biomolecular Engineering, Clemson University, 206 S. Palmetto Blvd., Clemson, SC 29634-0909 USA; 2 Department of Chemical & Biomolecular Engineering, University of Delaware, 590 Avenue 1743, Newark, DE 19713 USA

**Keywords:** Metabolic engineering, Biotechnology, Systems biology

## Abstract

Secretion levels required of industrial Chinese hamster ovary (CHO) cell lines can challenge endoplasmic reticulum (ER) homeostasis, and ER stress caused by accumulation of misfolded proteins can be a bottleneck in biomanufacturing. The unfolded protein response (UPR) is initiated to restore homeostasis in response to ER stress, and optimization of the UPR can improve CHO cell production of therapeutic proteins. We compared the fed-batch growth, production characteristics, and transcriptomic response of an immunoglobulin G_1_ (IgG_1_) producer to its parental, non-producing host cell line. We conducted differential gene expression analysis using high throughput RNA sequencing (RNASeq) and quantitative polymerase chain reaction (qPCR) to study the ER stress response of each cell line during fed-batch culture. The UPR was activated in the IgG_1_ producer compared to the host cell line and our analysis of differential expression profiles indicated transient upregulation of ATF6α target mRNAs in the IgG_1_ producer, suggesting two upstream regulators of the ATF6 arm of the UPR, ATF6β and WFS1, are rational engineering targets. Although both ATF6β and WFS1 have been reported to negatively regulate ATF6α, this study shows knockdown of either target elicits different effects in an IgG_1_-producing CHO cell line. Stable knockdown of *ATF6β* decreased cell growth without decreasing titer; however, knockdown of *WFS1* decreased titer without affecting growth. Relative expression measured by qPCR indicated no direct relationship between *ATF6β* and *WFS1* expression, but upregulation of *WFS1* in one pool was correlated with decreased growth and upregulation of ER chaperone mRNAs. While knockdown of *WFS1* had negative impacts on UPR activation and product mRNA expression, knockdown of *ATF6β* improved the UPR specifically later in fed-batch leading to increased overall productivity.

## Introduction

The bulk of industrialized production of therapeutics is accomplished using Chinese hamster ovary (CHO) cells^1–3^. Due to high demand for therapeutics, there is a need to identify strategies for overcoming the effects of misfolded proteins, or endoplasmic reticulum (ER) stress, resulting from recombinant protein overexpression and secretion. The unfolded protein response (UPR) serves as a primary quality control mechanism in CHO cells to enhance protein processing and reestablish ER homeostasis^2,3^. Changes in biomanufacturing media (e.g. nutrient ratio and availability), environmental parameters (e.g. pH, temperature, etc.), and infrastructure (e.g. reactor type) have been investigated as methods for controlling the CHO cell UPR^4–11^. Many CHO cell studies have focused on understanding and manipulating activation of Glucose-regulated protein 78/Binding immunoglobulin protein (GRP78/BiP) and downstream UPR transcription factors^12,13^, but other upstream regulators of the UPR also impact cell line performance.

In mammalian cells, the UPR is initiated when the chaperone GRP78/BiP binds to misfolded proteins after dissociating from complexes with one of the three canonical UPR activator proteins: Activating transcription factor 6, Inositol requiring enzyme 1, and Protein kinase R-like ER kinase (ATF6, IRE1, and PERK, respectively). Dissociation of GRP78/BiP from UPR activators triggers the primary transcription factors cleaved ATF6 alpha (ATF6α), spliced X box-binding protein 1 (XBP1s), and ATF4, respectively^14–19^. There are two isoforms of ATF6 (α and β) which are translocated to the Golgi and cleaved by site-1 and site-2 proteases (S1P and S2P, respectively)^20–22^. This cleavage event releases the amino-termini of ATF6α and ATF6β, each of which contains a basic leucine zipper (bZIP) DNA-binding motif. Primarily, ATF6α is translocated to the nucleus where it binds to ER stress response elements (ERSE I, CCAAT-N9-CCACG and ERSE II, ATTGG-N1-CCACG) in the promoter regions of target genes^23–25^. These target genes include, but are not limited to, *XBP1*, *GRP78/BiP*, isomerases and other chaperones needed within the ER^17,26,27^. The role of ATF6β in the UPR remains unclear.

Some literature suggests ATF6β is not significant^28^ while other literature suggests it acts as an antagonist of ATF6α through competitive promoter binding^20–22,29^. Knockdown of *ATF6β* improved fed-batch cell density and titer of a DG44-IgG_1_ CHO cell line^20^. In pancreatic β-cells, ATF6β is the lone transcription factor for *Wolframin (WFS1)*, and WFS1 inhibits ATF6α by targeting it for proteasomal degradation^30–32^. Thus, knockdown of *WFS1*, like knockdown of *ATF6β*, could also benefit CHO cell fed-batch production. Additionally, the gene *WFS1* was previously found upregulated in a highly proteolytic, low productivity CHO cell phenotype^33^, and knockdown of *WFS1* could increase CHO cell productivity.

In this study, we analyzed fed-batch culture samples of an IgG_1_-producing CHO cell line in comparison to its non-producing, parental CHO-K1 glutamate synthase knockout (GS KO) host cell line. We conducted a thorough analysis of dynamic gene expression changes using high throughput RNA sequencing (RNASeq) and quantitative polymerase chain reaction (qPCR) methods. During fed-batch culture, our findings show early induction of the UPR and protein processing in the IgG_1_ producer. Interestingly, multiple downstream targets of ATF6α with roles in glycoprotein folding and oxidative stress were transiently upregulated during fed-batch production. The RNASeq analysis also showed differential expression of two regulators of ATF6α, ATF6β and WFS1^20,30–33^. Based on these results, we targeted *ATF6β* and *WFS1* for CHO cell line engineering. To understand if each target had similar impacts on UPR management throughout the duration of a fed-batch culture, we created stable *ATF6β* and *WFS1* knockdown pools of the IgG_1_ producer using lentiviral delivered shRNA. We utilized qPCR to analyze the relationship between *ATF6β* and *WFS1* expressions as well as their effects on ATF6α target mRNA and product mRNA expression. Our results demonstrate stable knockdown of *ATF6β* as a useful strategy for ER stress management in an IgG_1_-producing cell line derived from a CHO-K1 GS KO host; however, knockdown of *WFS1* does not have the same effect. Overall, this study demonstrates value for transcriptomic analyses of CHO cell production throughout fed-batch culture and provides new insights for managing ER stress during recombinant protein production by manipulating and controlling the ATF6 pathway of the UPR.

## Materials and methods

### Culture conditions of CHO cells

Two proprietary CHO cell lines (Sigma-Aldrich) were used in this study: an IgG_1_ producer derived from CHOZN®GS^-/-^^34^ host and the non-producing host cell line CHOZN®GS^-/-^^34^. The glutamate synthase knockout host cell line (CHOZN®GS^-/-^) is the parental cell line for the IgG_1_ producer. Cell cultures of these cell lines and knockdown pools of the IgG_1_ producer were maintained according to the CHOZN® Platform Technical Bulletin (Sigma-Aldrich) with some modifications. Both passage and fed-batch cultures were grown in Erlenmeyer shake flasks (VWR®, Cat. No. 89095-262) at 100 rpm, 37° C, and 5% CO_2_. Each cell line was passaged at least once post-thaw to ensure similarly aged, healthy cultures for subsequent transcriptomic analysis of fed-batch samples. For passages, the culture volume corresponding to 0.5 × 10^6^ cells/mL was spun for 5 min at 500 rpm, aspirated, washed with 1X phosphate buffered saline (PBS), spun for 5 min at 500 rpm, aspirated, and resuspended in 25 mL of fresh EX-CELL® CD CHO Fusion media (Sigma-Aldrich, Cat. No. 14365C). Fed-batch cultures of the host, IgG_1_ producer, and knockdown pools of the IgG_1_ producer were run in biological triplicate. Fed-batch cultures were passaged in the same way, but resuspended in 30 mL of fresh EX-CELL® Advanced™ CHO Fed-batch media (Sigma-Aldrich, Cat. No. 14366C). Both passage and fed-batch medium for the host cell line were supplemented with L-glutamine (Corning, Cat. No. MT25005CI) to a final concentration of 5 mM. For the knockdown pools of the IgG_1_ producer, both passage and fed-batch mediums were supplemented with puromycin (Sigma-Aldrich Cat. No. P7255-25MG) to a final maintenance concentration of 1 µg/mL. During fed-batch, viable cell density (VCD) in units of × 10^6^ cells/mL and viability as a percentage were determined daily by hemocytometry using the trypan blue exclusion method. Integral of viable cell density (IVCD) in units of × 10^6^ cells*day/mL was determined by the following equation where t, t−1, and ∆t represent day t, the previous day, and the difference between days t and t−1, respectively^35^:

$$IVCD_{t} = IVCD_{t - 1} + \frac{{\left( {VCD_{t} + VCD_{t - 1} } \right)}}{{\left( {2 \times \Delta t} \right)}}$$

The IVCD on day 0, IVCD_0_, was equal to the VCD on day 0, VCD_0_. Feedings always took place after sampling. Feedings were carried out according to the CHOZN Platform Technical Bulletin. The host cell line was fed L-glutamine to a final concentration of 5 mM starting on day 3 and on all remaining odd days. All cell lines were fed an EX-CELL® Advanced™ CHO Feed 1 (Sigma-Aldrich, Cat. No. 24368C-1L) at 5% of the culture volume on day 3 and on all remaining odd days. Finally, all cell lines were fed D-(+)-Glucose solution (Sigma-Aldrich, Cat. No. G8769-100 mL) to a final concentration of approximately 4 g/L daily, once the glucose concentration dropped to 2 g/L. To limit bias due to lysis when measuring product titer, fed-batches were terminated when viability dropped below 70%.

### Preparation of fed-batch samples

Samples were always taken after determining VCD and viability and prior to feedings. Samples were prepared in the following manner: a cell culture volume not exceeding 5 × 10^6^ cells/mL was collected, spun down at 500 rpm for 5 min, aspirated, washed with 1X PBS, spun down at 500 rpm for 5 min, and aspirated. Cell pellets for RNA isolation were resuspended in 100 µL of RNALater™ Stabilization Solution (Thermo Fisher Scientific, Cat. No. AM7021). Cell pellets for mRNA samples were stored at −80° C until ready for extraction. Supernatants from the first spin were collected, spun down at maximum speed, and transferred to a new tube for glucose and IgG_1_ titer measurements using the Glucose Bio and IgG Bio assays for a Roche CustomBiotech Cedex Bio Analyzer (Roche Diagnostics, Mannheim, Germany), respectively. These glucose measurements were then used to determine glucose feedings. Calculations for specific productivity (q_P_) in units of pg/cell/day (pcd) were carried out two ways. Throughout fed-batch, q_P_ on day t (daily q_P_) was calculated as Titer_t_ / IVCD_t_. Overall q_P_ for each fed-batch assay was calculated as the slope of cumulative titer (mg/L) versus IVCD (× 10^6^ cells*day/mL)^35,36^. For the knockdown pools of the IgG_1_ producer, student’s paired *t* test was used to estimate statistical significance of titer and specific productivity relative to the Scramble control pool (see Table 1).

**Table 1:** Design matrix for shRNA experiment.

Target	Pool	Description
None^#^	Sham	Untreated (mock) control
	Scramble	Negative control
	GFP	Positive control
ATF6β	shATF6β.1*	Sigma MISSION® TRCN0000013720
	shATF6β.2•	Sigma MISSION® TRCN0000013721
	shATF6β.3***	Sigma MISSION® TRCN0000013722
	shATF6β.4•	Sigma MISSION® TRCN0000075444
	shATF6β.5***	Sigma MISSION® TRCN0000075446
WFS1	shWFS1.1•	Sigma MISSION® TRCN0000364298
	shWFS1.2•	Sigma MISSION® TRCN0000364365
	shWFS1.3•	Sigma MISSION® TRCN0000376390
	shWFS1.4•	Sigma MISSION® TRCN0000255639
	shWFS1.5***	Sigma MISSION® TRCN0000364297

^#^For the Sham control, the IgG_1_-producing cell line went through a mock transduction and selection process where neither lentivirus nor puromycin were added to the pool. For the Scramble control, the shRNA sequence is a randomized sequence with no intended target. The GFP control had no shRNA sequence and was used as a positive control to refine the spinoculation protocol.

*Did not survive selection.

•Did not knockdown the respective target in this study.

***Confirmed knockdown of the respective target in this study.

### RNA extractions

Extractions of RNA were carried out using the Qiagen Rneasy Plus Mini Kit (Qiagen, Cat. No. 74136). Extractions were performed according to the manufacturer’s protocol for “Purification of Total RNA from Animal Cells” with the following changes: samples were prepared as in "Preparation of fed-batch samples" (Step 1), the lysate was passed through a 21-gauge needle at least 5 times (option 3c of Step 3), molecular grade ethanol was added to the flow through rather than 70% ethanol (Step 5), the RNeasy spin column was spun in a new 2 mL collection tube (optional Step 10) , and Dnase/Rnase-free water was heated to 37° C and allowed to incubate on the column for 5 min prior to elution (Step 11). Quantification of RNA was performed by measuring absorbance at 260 and 280 nm using a nanospectrometer. Multiple aliquots of 100 ng/µL were prepared and stored at −80° C until ready for analysis.

### Processing of RNA sequencing

For the fed-batch assays of the host and IgG_1_ producer, biological triplicate samples for days 0, 1, 3, and 5 were subjected to high throughput RNA sequencing. Because multiple aliquots were made at the time of RNA extraction, the same RNA was used for both qPCR and the transcriptomic analysis. Concentrations of RNA and RNA Integrity Numbers (RIN) were determined by Qubit fluorometer (Invitrogen) and 2100 Bioanalyzer (Agilent), respectively. Library preparation and paired-end sequencing were performed by Novogene Corporation Inc. (Sacramento, CA) using the Illumina NovaSeq 6000 platform. Raw read processing software was as follows: Trimmomatic (0.36) for adapter removal, FastQC (0.11.8) for quality control analysis of raw reads, STAR (2.6.0a) for alignment of the raw reads to the CHO CriGri-PICR reference genome assembly (GCF_003668045.1_CriGri-PICR), and HTSeq (0.10.0) for generation of the read count tables^37–41^. One replicate of the day 1 host samples was lost during library preparation and not included in the analysis. To generate read counts for the three product mRNAs, the CriGri-PICR genome file was amended to include two additional gene sequence files for the products: IgG_1_ light chain (IgG_1_ LC) and IgG_1_ heavy chain (IgG_1_ HC). Sequencing data has been deposited in the National Center for Biotechnology Information’s Gene Expression Omnibus database, URL accession number GSE217637.

### Differential gene expression analysis

Principal component analysis (PCA) and differential expression testing, based on the Wald statistical pairwise test, were conducted using DESeq2 (1.34.0) software^42^. The PCA of the dataset confirmed sample replicates of both the control (host) and treatment (IgG_1_ producer) groups clustered accordingly (Supplemental Figure S1). Fragments per kilobase pair million (FPKM) were calculated using the built-in function in DESeq2 software based on the known lengths of target genes. For all pairwise differential gene expression analysis, the absolute value of the log2 fold change (log2FC) cutoff was set to 0.5 and an adjusted p-value cutoff of 0.01 was used, as identified by an elbow of all DEGs versus various adjusted p-value cutoffs (Supplemental Figure S2). In order to observe how each cell line copes with fed-batch culture over time, differential expression analysis was conducted between a fed-batch culture time point and the respective day 0 time point for each cell line (e.g. IgG_1_ producer day 1 vs. IgG_1_ producer day 0). Likewise, in order to observe transcriptomic variations in the IgG_1_ producer, differential expression analysis was conducted in the IgG_1_ producer versus the host cell line at a respective time point (e.g. IgG_1_ producer day 1 vs. Host day 1). Gene lists for specific pathways were compiled using the Kyoto Encyclopedia of Genes and Genomes (KEGG) database for *Cricetulus griseus* (Chinese hamster) and were used to render heatmaps illustrating log2 fold change values relative to controls. Primary focus was on the KEGG pathways Protein Processing in the ER (including the UPR), Peroxisome, and Glutathione Metabolism.

### Quantitative polymerase chain reaction

Biological triplicate reactions were carried out on either a Bio-Rad CFX Connect Real-Time Thermal Cycler or a Bio-Rad CFX Opus Real-Time PCR System. For each biological replicate, qPCR was carried out with 100 ng of total RNA using the qScript 1-Step SYBR Green qRT-PCR Kit (QuantaBio, Cat. No. 95054-946). All primer efficiencies measured between 90–110%. Each total reaction volume was 10 µL supplemented with 0.4 µM gene-specific primers, provided in Supplemental Table S1. The reaction conditions were as follows: 10 min at 50° C to activate the Taq polymerase, denaturation of 95° C for 10 min., followed by 40 cycles of 95° C for 15 s, annealing at 60° C for 25 s, extension at 72° C for 30 s. Melting curve analysis was used to check for primer specificity, and no reverse transcriptase and no template controls were used to check for genomic DNA and primer-dimers, respectively. The housekeeping gene was β-actin^43^. The ∆∆Ct method was used for computing relative expression levels^44^. The relative expression level of target mRNAs during fed-batch for the host cell line and the IgG_1_ producer was defined by Eqs. 1, 2 for a gene of interest (GOI) in a sample from day *t.* The relative expression level of target mRNAs during fed-batch for the knockdown pools of the IgG_1_ producer was defined by Eqs. 1, 3 for a GOI in a sample from day *t.*

1 $$Expression level = 2^{ - \Delta \Delta Ct}$$

2 $$\Delta \Delta Ct = \left[ {\left( {Ct_{Sample, day t}^{GOI} - Ct_{Host, day 0}^{GOI} } \right) - \left( {Ct_{Sample, day t}^{\beta - actin} - Ct_{Host, day 0}^{\beta - actin} } \right)} \right]$$

3 $$\Delta \Delta Ct = \left[ {\left( {Ct_{Sample, day t}^{GOI} - Ct_{Scramble, day 0}^{GOI} } \right) - \left( {Ct_{Sample, day t}^{\beta - actin} - Ct_{Scramble, day 0}^{\beta - actin} } \right)} \right]$$

After propagating standard deviation for ∆∆Ct values, error for relative expression levels was calculated as detailed previously^44^. Student’s unpaired *t* test was used to estimate statistical significance of ∆∆Ct values of the IgG_1_ producer with respect to the host cell line on the same day (Fig. 4 and Supplemental Table S2). Student’s paired *t* test was used to estimate statistical significance of ∆∆Ct values of knockdown pools with respect to the Scramble pool (see Table 1) on the same day (Fig. 8 and Supplemental Table S3).

### Lentiviral vector packaging and titration

Transfer plasmids were packaged with the psPAX.2 packaging plasmid (Addgene #12260) and the pVSV.G envelope plasmid (Addgene #8454). For each of the knockdown targets (*ATF6β* and *WFS1*), bacterial stocks of five different validated MISSION® shRNA sequences in a pLKO.1-puro plasmid were purchased from Sigma-Aldrich. The MISSION® shRNA sequences used in this study were relabeled for clarity during experiments, and both identifiers are listed in Table 1.

A randomized, non-targeting (Scramble) shRNA sequence (Addgene #1864) was used as a negative control to test for off-target effects. A Green fluorescent protein (GFP) (Addgene #17448) transfer plasmid was used as a positive control. All lentiviral plasmids contained a puromycin selection marker. To improve yields, all plasmids were transformed into One Shot® Stbl3™ Chemically Competent *Escherichia coli* cells (Invitrogen Cat. No. C7373-03). The PureYield™ Plasmid Maxiprep System (Promega Cat. No. A2393) was used to elute all plasmid DNA prior to sterilization and concentration via ethanol precipitation. Lentivirus was produced following routine methods^45^. Briefly, lipofectamine and DNA master mixes for each shRNA sequence were prepared using Opti-MEM™ Reduced Serum Medium (Thermo-Fisher Cat. No. 31985070), combined, and allowed to incubate at room temperature for 30 min. After aspirating the growth medium, the transfection mix was dispersed across 5 × 10^6^ HEK293 cells and incubated for 1 min prior to adding 10 mL of fresh growth medium. The growth medium was replaced daily for another 3 days. The growth medium was collected as viral supernatant on days 4 and 5 and stored in the dark at 4° C. The viral supernatant was concentrated using Amicon Ultra-15 filters NMW 100 kD (Millipore Cat. No. UFC910008). Aliquots of lentivirus were stored at −80° C until use. Collected lentivirus was titered via qPCR using the Applied Biological Materials qPCR Lentivirus Titration Kit (Cat. No. LV900) (Supplemental Figure S3).

### Spinoculation, selection, and cryostocking of pools

For the shRNA study, all knockdown pools and controls were derived from the IgG_1_-producing CHO cell clone (Sigma-Aldrich). We adapted the spinoculation protocol based on “Infection Protocol for Recombinant Lentivirus” and “Spinoculation Protocol” (SignaGen® Laboratories, Rockville, MD; Sigma-Aldrich). As a negative control, the IgG_1_ cell line was grown in a mock transduction and selection protocol (Sham) where no lentivirus or puromycin was used. The GFP lentivirus was used to adjust the spinoculation protocol and refine the ratio of viral particles to cells. At least 1 × 10^6^ virus particles were used to transduce 1 × 10^6^ cells with 8 µg/mL polybrene (Sigma-Aldrich, Cat. No. TR-1003-G). Separate transductions were carried out for each of the lentiviral vectors. Transduction cell suspensions were gently mixed and incubated for 10 min at room temperature prior to being centrifuged at 800xg for 30 min. The viral supernatant was aspirated off the cell pellet and bleached. The cell pellet was resuspended in 1 mL of fresh EX-CELL® CD CHO Fusion media (Sigma-Aldrich, Cat. No. 14365C) and transferred to a non-treated 24-well plate. After three days, transduced cultures were passaged as 1:2 splits in the presence of 8 µg/mL of puromycin (Sigma-Aldrich, Cat. No. P7255-25MG). Puromycin, at a final concentration of 8 µg/mL, was used for selection for all subsequent passages. After two passages, transduced cells were passaged as 1:2 splits into a final volume of 2 mL in a non-treated 6-well plate. Passages were carried out as 1:2 splits every 3 or 4 days until transduced cultures achieved 90% viability as determined by hemocytometry using the trypan blue exclusion method. During selection, passages in well-plates were maintained in a stationary environment (37˚C and 5% CO_2_) to reduce transduction-related stress. After achieving 90% viability, enough cells were passaged such that cells could be passaged into Erlenmeyer shake flasks (VWR®, Cat. No. 89095-262) in a volume of 25 mL at 0.5 × 10^6^ cells/mL and spun at 100 rpm, 37° C, and 5% CO_2_. Cells were passaged twice in shake flasks, under the same conditions, before cryostocking. Cryostocks were prepared in EX-CELL® CD CHO Fusion media supplemented with 10% DMSO and frozen in Mr. Frosty™ Freezing Containers, prior to storage in liquid nitrogen.

### Assessment of shRNA knockdown pools

For the shRNA study, all selected knockdown pools were grown in a single replicate fed-batch assay along with the Sham control. To confirm knockdown of *ATF6β* and *WFS1* in selected pools, a single day was used for RNA extraction and qPCR analysis. Based on the initial fed-batch assays of the host cell line and the IgG_1_ producer, day 5 was a logical choice given the UPR activation timeline and the reported roles of ATF6β and WFS1^20–22,29–33^. To determine percent knockdown, qPCR was run as described in “Quantitative polymerase chain reaction” except samples were analyzed in technical triplicate, and relative expression levels were calculated as described in “Quantitative polymerase chain reaction” except Ct values for the Sham and/or Scramble pool on day 5 were used as the calibrator (rather than day 0) (Fig. 5 and Supplemental Figure S8).

## Results

### The IgG_1_ producer exhibits reduced growth and high titer and cell specific productivity

**Figure 1:**
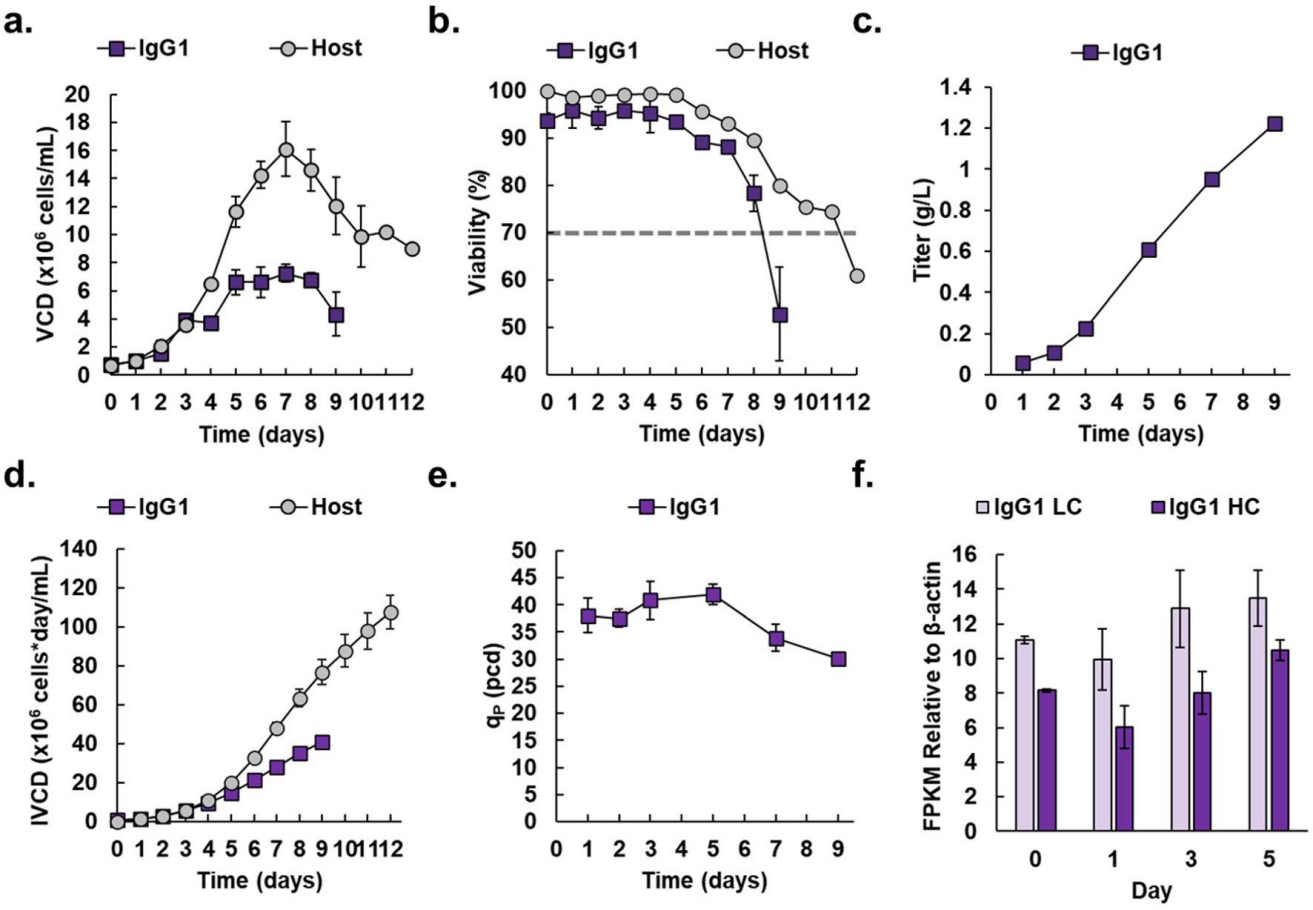
Growth characteristics of protein producing cell lines compared to a non-producing host cell line. (**a**) Viable cell density (VCD) in × 10^6^ cells/mL for the IgG_1_-producer (purple squares) and host (gray circles). (**b**) Viability as a percentage for the IgG_1_ producer (purple squares) and host (gray circles). Dashed line represents 70% viability (**c**) Titer in g/L for the IgG_1_ producer (purple squares). Data which were lower than our detection limit were omitted. (**d**) Integral of VCD (IVCD) in × 10^6^ cells*day/mL for the IgG_1_ producer (purple squares) and host (gray circles). (**e**) Specific daily productivity (q_P_) in pcd throughout fed-batch culture for the IgG_1_ producer (purple squares). Data which were lower than our detection limit were omitted. (**f**) Fragments per kilobase pair Million (FPKM) relative to β-actin for product mRNAs IgG_1_ light chain (IgG_1_ LC, light purple bars) and IgG_1_ heavy chain (IgG_1_ HC, dark purple bars). Data which were lower than our detection limit were omitted. All data are shown as average ± SD (*N* = 3).

We initially compared the fed-batch production characteristics of the IgG_1_ producer to its parental, non-producing host cell line. In fed-batch shake flasks, the IgG_1_ producer reached a maximum VCD of 7.3 ± 0.61 × 10^6^ cells/mL, while the host cell line reached a higher maximum VCD of 16 ± 2.0 × 10^6^ (Fig. 1a). Viability for the IgG_1_-producing cell line did not drop below 70% until day 9 of fed-batch culture, while viability did not drop below 70% until day 12 for the host cell line (Fig. 1b). For the IgG_1_ producer, reduced VCD and shortened fed-batch culture suggest metabolic burden due to protein production. The IgG_1_ producer reached a titer of 1.2 ± 0.031 g/L, an IVCD of 41 ± 1.1 × 10^6^ cells*day/mL by day 9, and a peak daily q_P_ of 42 ± 2.0 pcd on day 5 (Fig. 1c, d, e, respectively). The slope of the cumulative titer versus IVCD showed the overall q_P_ for the IgG_1_ producer was 30. ± 0.89 pcd (Supplemental Figure S4). Samples from days 0, 1, 3, and 5 of fed-batch culture were subjected to RNASeq. To understand the contribution of mRNA expression levels towards the observed fed-batch titer and productivity, we calculated the FPKM of each of the product mRNAs relative to the housekeeping gene β-actin (Fig. 1f). The IgG_1_ producer exhibited higher expression of light chain compared to heavy chain, at least 9.93-fold and 6.03-fold higher relative to β-actin, respectively, which is a common characteristic of highly productive IgG cell lines^46–48^.

### Transient upregulation of UPR and downstream mRNAs in the IgG_1_ producer

**Figure 2:**
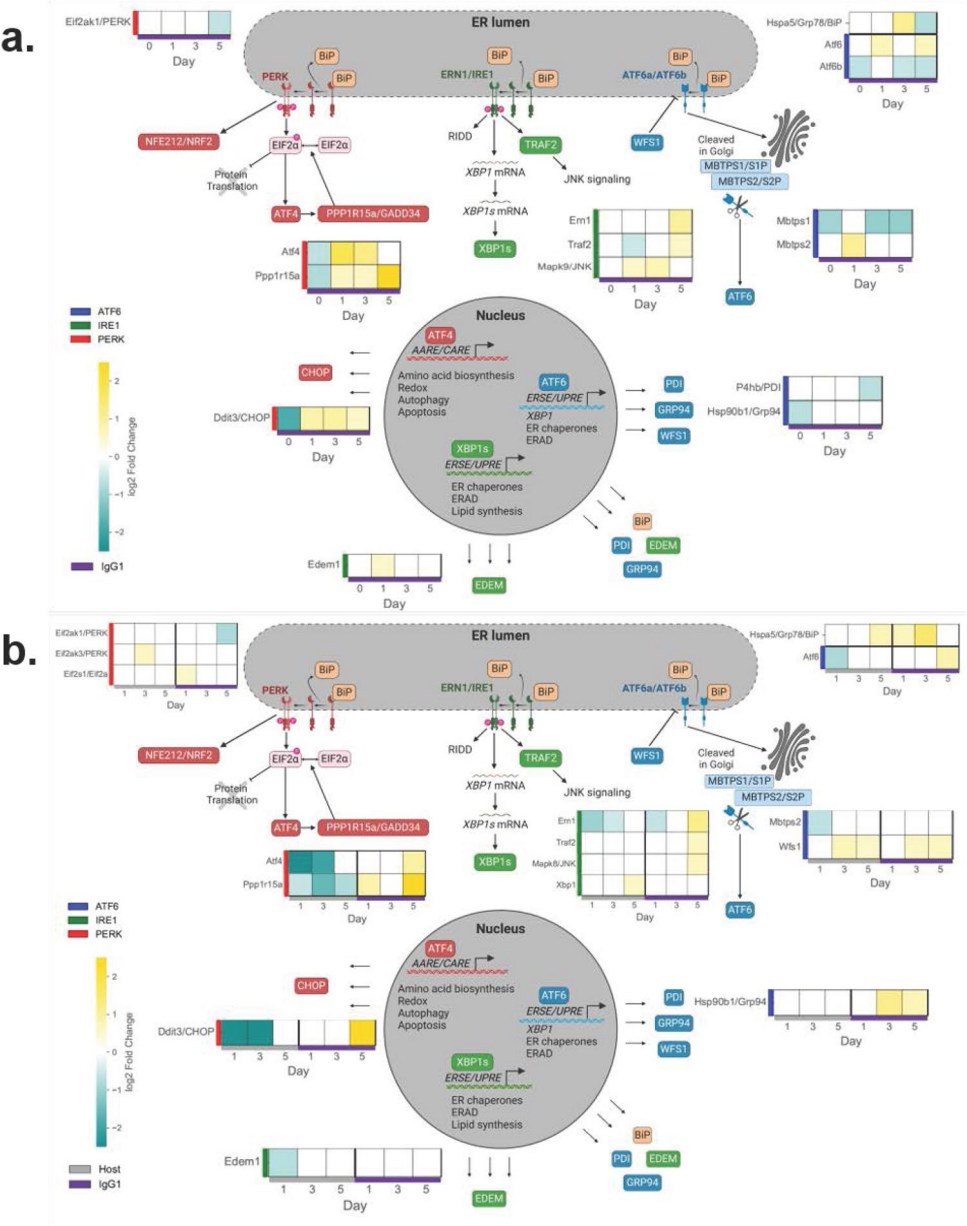
Heatmaps of differentially expressed UPR mRNAs. Differentially expressed transcripts have an absolute value of the log2FC greater than 0.5 and an adjusted *P*-value less than 0.01 (*N* = 3). Transcripts which are upregulated are shown in gold, and transcripts which are downregulated are shown in cyan. (**a**) For UPR mRNAs, representations of log2FC are shown in individual squares representing a specific day in fed-batch culture versus the host cell line. The bottom legend shows samples for the IgG_1_ producer (purple). Left legend shows transcripts as organized by UPR pathways ATF6 (navy), IRE1 (green), and PERK (red). (**b**) For UPR mRNAs, representations of log2FC are shown in individual squares representing a specific day in fed-batch culture versus day 0 for the respective cell line. The bottom legend shows samples for the host cell line (gray) and the IgG_1_ producer (purple). Left legend shows transcripts as organized by UPR pathways ATF6 (navy), IRE1 (green), and PERK (red). The bottom legend shows samples for the host cell line (gray) and the IgG_1_ producer (purple). Figure created with BioRender.com.

**Figure 3:**
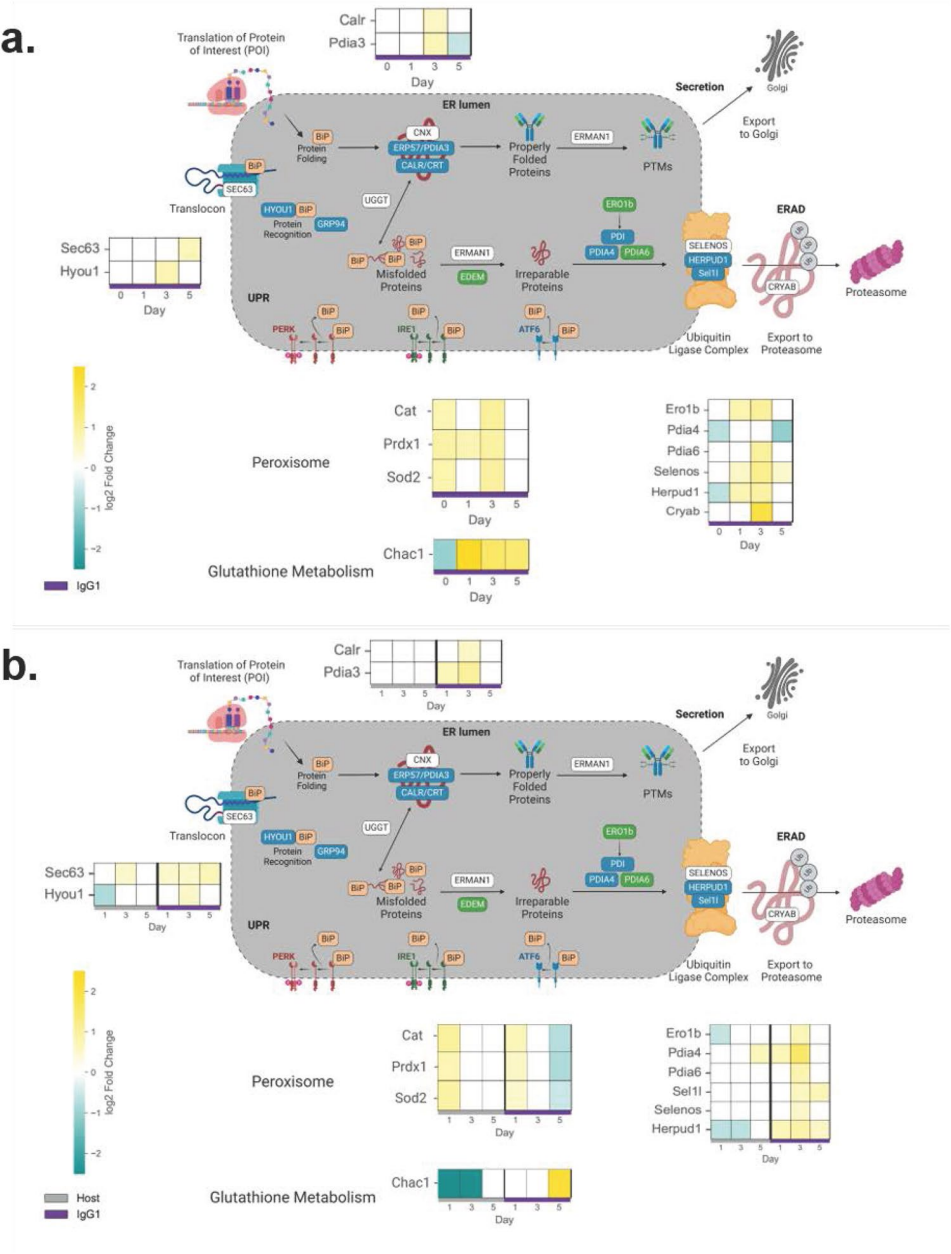
Heatmaps of differentially expressed protein processing mRNAs. Differentially expressed transcripts have an absolute value of the log2FC greater than 0.5 and an adjusted p-value less than 0.01 (*N* = 3). Transcripts which are upregulated are shown in gold, and transcripts which are downregulated are shown in cyan. (**a**) For protein processing mRNAs, representations of log2FC are shown in individual squares representing a specific day in fed-batch culture versus the host cell line. The bottom legend shows samples for the IgG_1_ producer (purple). (**b**) For protein processing mRNAs, representations of log2FC are shown in individual squares representing a specific day in fed-batch culture versus day 0 for the respective cell line. The bottom legend shows samples for the host cell line (gray) and the IgG_1_ producer (purple). Figure created with BioRender.com.

Because the IgG_1_ producer in this study has high specific productivity, it was hypothesized this cell line would exhibit early activation of the UPR during fed-batch culture. Accordingly, early timepoints of days 0, 1, 3, and 5, were selected for RNASeq analysis. Although growth and viability were similar for both cell lines early (up to day 3) in fed-batch culture, transient upregulation of UPR and downstream mRNAs in the IgG_1_ producer suggests transcriptional adaptation for protein production to maintain ER homeostasis (Figs. 2, 3 and Supplemental Figures S5, 6). When compared to the host cell line, the mRNA for *GRP78/BiP* was upregulated on day 3 in the IgG_1_ producer at a maximum of 2.1-fold (Fig. 2a and Supplemental Figure S5a), indicating the UPR is activated early in the IgG_1_ producer compared to the host cell line. The PERK pathway is strongly upregulated in the IgG_1_ producer, as indicated by the profiles for *ATF4*, *DNA damage-inducible transcript 3/C/EBP homologous protein (Ddit3/CHOP)*, and *PPP1R15A/GADD34* with maximums of 3.2, 1.9, and 5.7-fold, respectively. The mRNA *MBTPS1* corresponds to S1P (needed to cleave ATF6 isoforms) and is primarily downregulated in the IgG_1_ producer, particularly at later time points with a maximum of 2.1-fold. The ATF6β isoform has previously been shown to competitively inhibit promoter binding of ATF6α^21,22,29^. Given knockdown of *ATF6β* has been successful in other IgG_1_-producing CHO cell cultures^20^, it is notable *ATF6β* is mostly downregulated in the IgG_1_ producer with a maximum of 1.6-fold.

When compared to day 0, mRNAs in all three of the canonical UPR pathways are downregulated early in the host cell line unlike the IgG_1_ producer (Fig. 2b and Supplemental Figure S5b). For example, the PERK pathway was downregulated in the host with *ATF4* and *CHOP* mRNAs expressed at maximums of 6.5 and 7.0-fold, respectively (Fig. 2b and Supplemental Figure S5b). The IgG_1_ producer upregulated *GRP78/BiP* by day 1 at 1.6-fold (maximum 2.8-fold on day 3) while the host cell line upregulated this mRNA by day 5 at 1.6-fold. The IgG_1_ producer also upregulated the chaperone *GRP94* by day 3 at a maximum of 2.3-fold. We also see several mRNAs throughout all three of the UPR pathways are upregulated in the IgG_1_ producer by day 5. For example, *CHOP* and *Protein phosphatase 1 regulatory subunit 15A/ growth arrest and DNA damage-inducible protein (PPP1R15A/GADD34)* mRNAs were upregulated at maximums of 5.3 and 5.7-fold, respectively. In other cell types (e.g. rodent β cells and human lymphocytes), the protein WFS1 targets ATF6α for degradation in the proteasome^20,30,31^. In our CHO cell study, the mRNA (and presumably the protein) *WFS1* is upregulated in both cell lines starting at day 3 with expressions of 1.6 and 1.5-fold in the host and IgG_1_ producer, respectively.

As most UPR mRNAs (particularly *GRP78/BiP*) were upregulated by day 3 in the IgG1 producer, we identified corresponding protein processing transcripts with a similar expression profile (Fig. 3 and Supplemental Figure S6). Glycoprotein folding chaperones *Calreticulin (CRT)* and *Protein disulfide isomerase 3 (PDIA3)* exhibited peak expression on day 3 in the IgG_1_ producer, similarly to the luminal chaperone *GRP78/BiP*. Compared to the host cell line, maximum expressions of *CRT* and *PDIA3* in the IgG_1_ producer were 1.5 and 1.6-fold, respectively (Fig. 3a and Supplemental Figure S6a), while maximum expressions of *CRT* and *PDIA3* were 1.5 and 2.0-fold, compared to day 0, respectively (Fig. 3b and Supplemental Figure S6b). Many other mRNAs exhibited similar peak fold expressions on day 3 in the IgG_1_ producer including *ER oxidoreductin 1 beta (ERO1b)*, *PDIA4, PDIA6,* and *Hypoxia up-regulated protein 1 (HYOU1)* (Fig. 3 and Supplemental Figures S6a, b). The corresponding proteins of these upregulated mRNAs play roles in ERAD and oxidative protein folding^49^. As such, we also analyzed transcripts involved in oxidative protein folding (e.g., the Peroxisome and Glutathione Metabolism KEGG pathways (Fig. 3 and Supplemental Figures S6c, d). We found the mRNA *ChaC glutathione specific gamma-glutamylcyclotransferase 1 (CHAC1)* displayed a similar expression profile to that of *ATF4, CHOP,* and *Homocysteine-responsive endoplasmic reticulum-resident ubiquitin-like domain member 1* (*HERPUD1)* in both cell lines*,* while the mRNAs for *Catalase (CAT), Peroxiredoxin-1 (PRDX1),* and *Superoxide dismutase 2 (SOD2)* displayed transient increases on day 3 in the IgG_1_ producer, consistent with other CHO cell studies^4–8,18,20,24,50–57^. Collectively, these mRNAs which are upregulated on day 3 are involved in glycoprotein folding, ERAD, and oxidative stress and are transcribed through the individual or combined action of ATF6α and XBP1s^25,28,58–61^. Because *ATF6β* and *WFS1* have each been identified as negative regulators of ATF6α ^20–22,29–32^, differential expression of *ATF6β* and *WFS1* in the IgG_1_ producer suggests unique regulation of, and reliance on, ATF6α in this cell line.

### Activation of ATF6α drives distinct cell-line specific expression profiles

**Figure 4:**
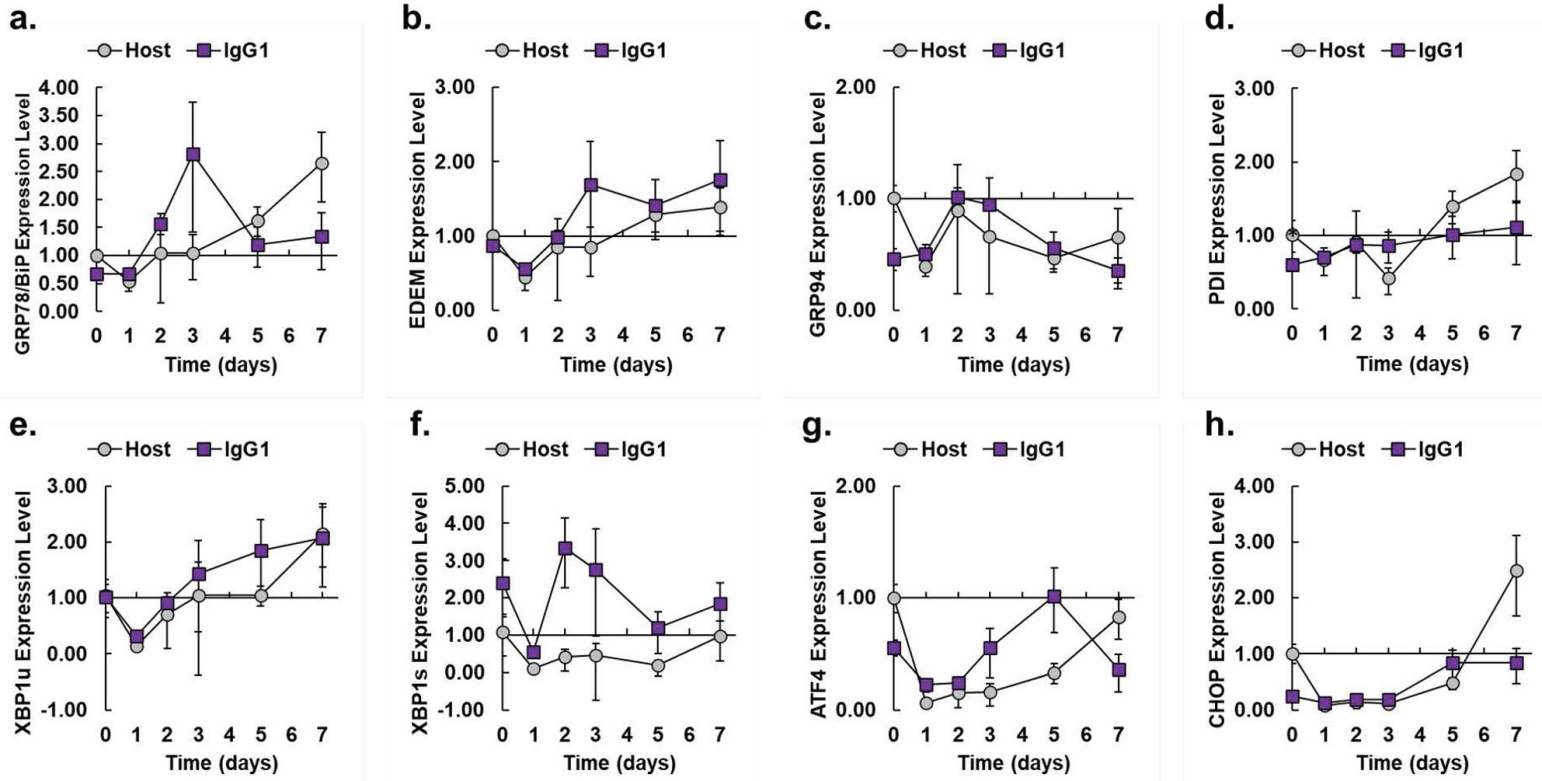
Expression level of UPR biomarkers in host and IgG_1_ producer as measured by qPCR. Expression levels of UPR target genes (**a**) *GRP78/BiP* (**b**) *GRP94* (**c**) *PDI* (**d**) *EDEM* (**e**) *XBP1u* (**f**) *XBP1s* (**g**) *ATF4* (**h**) *CHOP* vs days of fed-batch culture for each of the cell lines (IgG_1_ producer, purple squares; Host, gray circles). Calculations are relative to day 0 levels for the host cell line. The β-actin gene was used as the reference gene. After propagating standard deviation for ∆∆Ct values, error for relative expression levels was calculated as detailed previously (*N* = 3)^44^. Unpaired Student’s *t*-test results for Fig. 4 are provided in Supplemental Table S2.

To validate the RNAseq results, we chose a subset of common UPR markers for qPCR analysis (Supplemental Table S1). The ∆∆Ct method was used to calculate the expression level of target mRNAs relative to the reference gene β-actin and normalized to the host cell line on day 0 (Fig. 4 and Supplemental Table S2). The qPCR expression profiles for *GRP78/BiP*, *GRP94*, *PDI*, *ATF4*, and *CHOP* are consistent with the respective FPKM obtained from the RNASeq data (Compare Fig. 4 and Supplemental Figure S7).

Comparable to the RNASeq data, several UPR genes were transiently upregulated in the IgG_1_ producer relative to the host cell line, confirming the UPR is activated early in fed-batch production. During fed-batch, the IgG_1_ producer exhibited maximum expressions of *GRP78/BiP* and *ER degradation-enhancing alpha-mannosidase-like 1 (EDEM)* at 2.8 and 1.8-fold, respectively (Fig. 4a, b, respectively). Expression of chaperones *GRP94* and *P4HB/PDI* were similar for both cell lines (Fig. 4c, d, respectively). The IgG_1_ producer also transiently upregulated the unspliced and spliced forms of *XBP1* at maximum expressions of 2.1 and 3.3-fold, respectively (Fig. 4e, f, respectively). Compared to the host, the IgG_1_ producer exhibited increases in *ATF4* and *CHOP* expressions at least through day 5 (Fig. 4g, h, respectively). The expression profiles of *GRP78/BiP, EDEM*, *XBP1u*, and *XBP1s* in the IgG_1_ producer indicated distinct dynamics of the UPR where the IgG_1_ producer achieved peaks on day 3 for multiple protein processing mRNAs. In particular, the mRNAs *GRP78/BiP* and *EDEM* are targets of the individual or combined action of ATF6α and XBP1s, while *XBP1u* is a known target of ATF6α, supporting the results shown in Figs. 2, 3 and emphasizing a dependency on ATF6α activity in the IgG_1_ producer^25,28,58–61^. Therefore, we hypothesized knockdown of negative regulators, *ATF6β* and *WFS1*, would improve ATF6α activation, increase expression of ATF6α activated mRNAs and, subsequently, increase CHO cell specific productivity.

### Expressions of ATF6β and WFS1 stably knocked down by shRNA in the IgG_1_ producer

**Figure 5:**
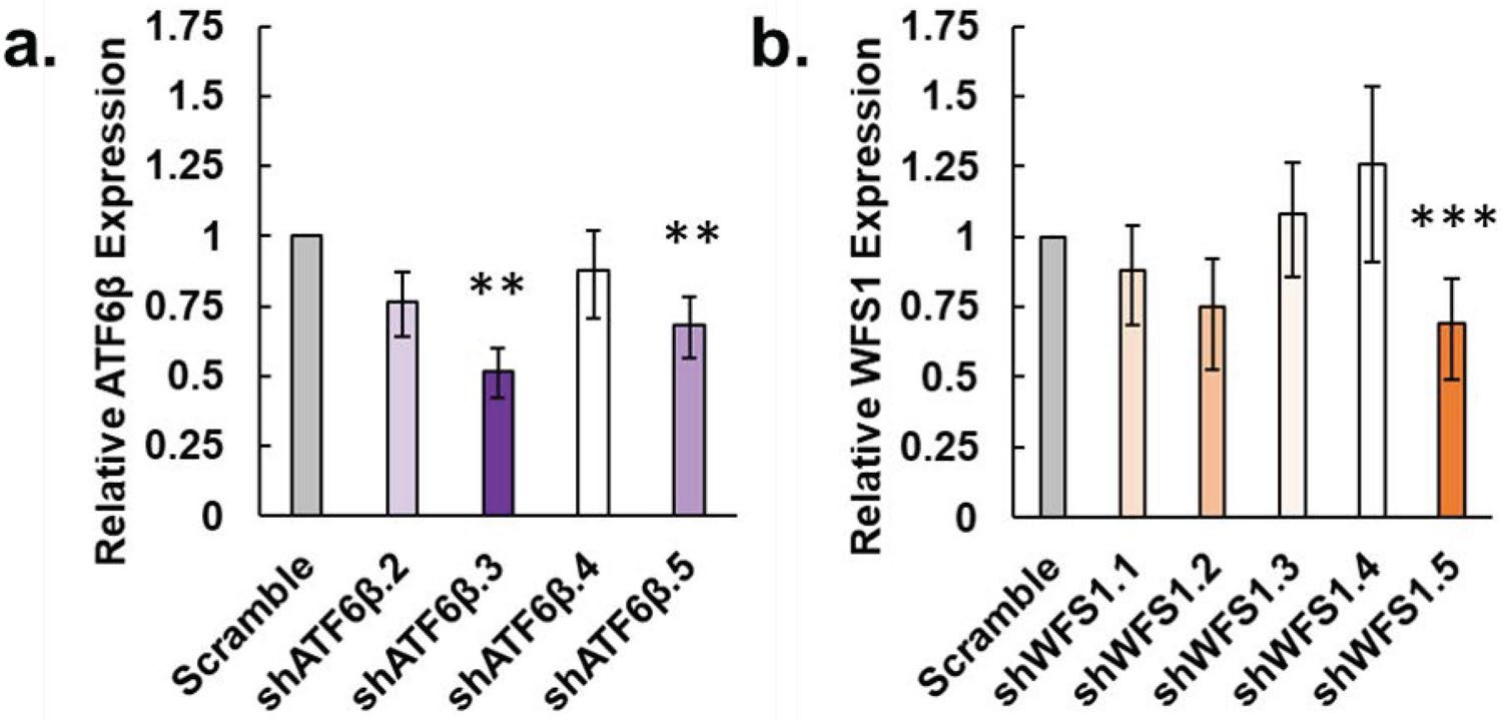
Relative expression of *ATF6β* and *WFS1* in selected IgG_1_-producing pools on day 5 of fed-batch. (**a**) Expression of ATF6β relative to the scramble control (gray bar) for shATF6β knockdown pools (purple bars). (**b**) Expression of WFS1 relative to the scramble control (gray bar) for shWFS1 knockdown pools (orange bars). All calculations are relative to day 5 levels. The β-actin gene was used as a housekeeping gene. After propagating standard deviation for ∆∆Ct values, error for relative expression levels was calculated as detailed previously (*N* = 3, **P < 0.05 Students paired t-test for two sample means with Scramble as the reference, ***P > 0.05 Students paired t-test for two sample means with Sham as the reference)^44^.

Lentivirus is an efficient RNA interference (RNAi) method for stable integration of shRNAs^45^. We developed stable (rather than transient) *ATF6β* and *WFS1* knockdown pools of the IgG_1_-producing CHO cell line to ensure expression was knocked down for the duration of fed-batch assays. A negative (Sham) control went through the transduction process with no lentivirus or puromycin added, and a non-targeting (Scramble) control was used to test for off-target effects. A single replicate fed-batch assay was used as an assessment of the selected knockdown pools in comparison to the Sham and Scramble controls. Relative *ATF6β* and *WFS1* expression was measured by qPCR in technical triplicate on RNA extracted from day 5 samples to determine which selected pools were successfully knocked down for either target (Fig. 5).

The Scramble pool did not exhibit knockdown of either *ATF6β* or *WFS1* in calculations relative to the Sham control, indicating the Scramble pool is an effective control (Supplemental Figure S8). Relative to the Sham control, the shATF6β.3 pool exhibited the highest percentage knockdown of *ATF6β* at 57% (Supplemental Figure S8a), and the shWFS1.5 pool exhibited the highest percentage knockdown of *WFS1* at 37% (Supplemental Figure S8b). Ultimately, we continued our analyses using pools which exhibited at least 30% knockdown of *ATF6β* or *WFS1* relative to the Scramble control (Fig. 5a, b, respectively). Of the pools knocked down for *ATF6β*, shATF6β.3 and shATF6β.5 exhibited 48 and 32% knockdown, respectively, relative to the Scramble control (Fig. 5a). Likewise, of the pools knocked down for *WFS1*, shWFS1.5 exhibited 31% knockdown (Fig. 5b).

### Knockdown of ATF6β, but not WFS1, decreases growth of IgG_1_ producer without decreasing titer

**Figure 6:**
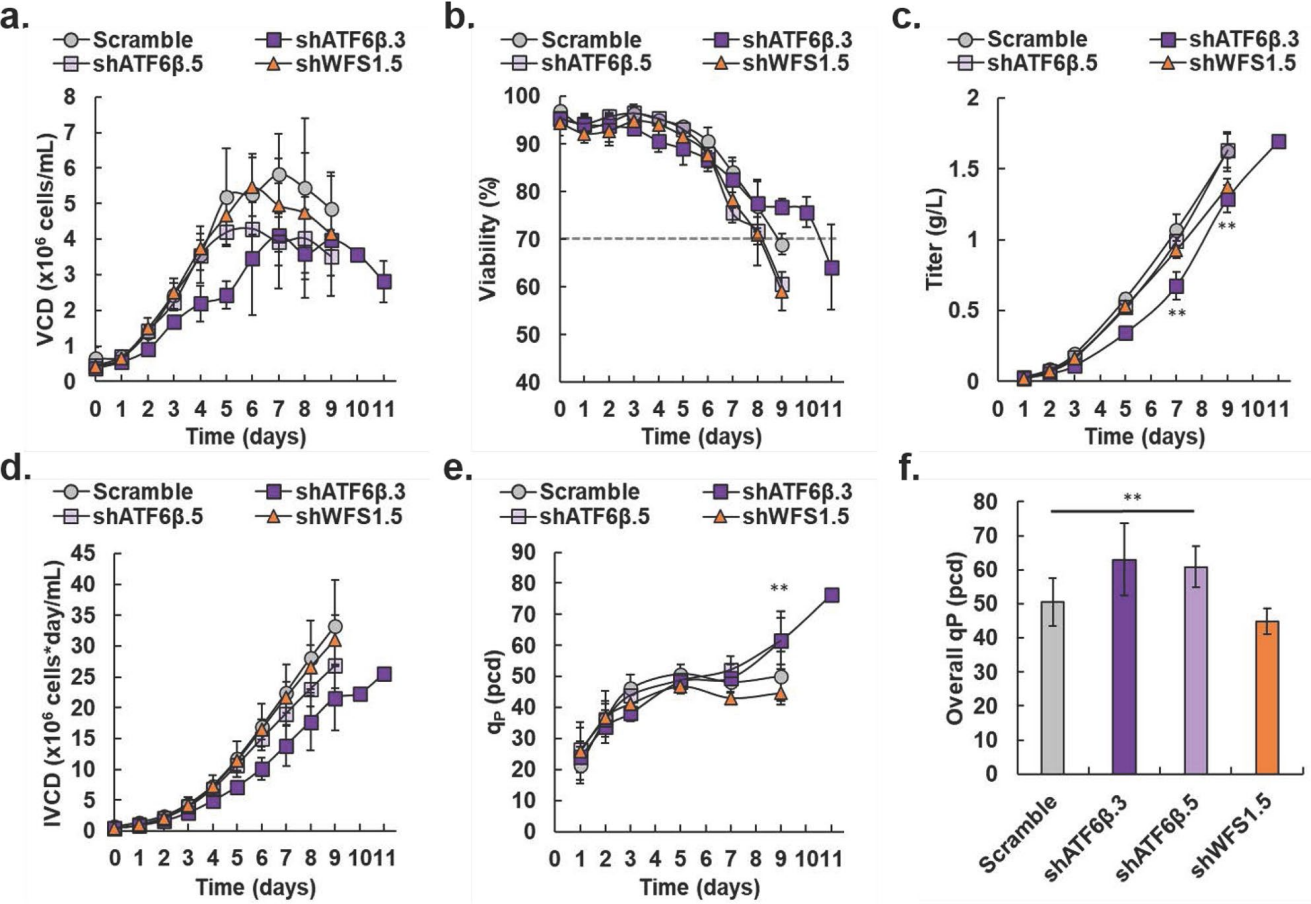
Growth characteristics of IgG_1_-producing knockdown pools compared to scramble control pool. (**a**) Viable cell density (VCD) in × 10^6^ cells/mL for scramble control (gray circles), shATF6β.3 (dark purple squares), shATF6β.5 (light purple squares), and shWFS1.5 (orange triangles). (**b**) Viability as a percentage for scramble control (gray circles), shATF6β.3 (dark purple squares), shATF6β.5 (light purple squares), and shWFS1.5 (orange triangles). Dashed line represents 70% viability. (**c**) Titer in g/L for scramble control (gray circles), shATF6β.3 (dark purple squares), shATF6β.5 (light purple squares), and shWFS1.5 (orange triangles). On day 7, ** is for the shATF6β.3 pool. On day 9, ** is for both the shATF6β.3 and shWFS1.5 pools. Data which were lower than our detection limit were omitted. (**d**) Integral of VCD** (**IVCD) in × 10^6^ cells*day/mL for, scramble control (gray circles), shATF6β.3 (dark purple squares), shATF6β.5 (light purple squares), and shWFS1.5 (orange triangles). (**e**) Specific daily productivity (q_P_) in pcd throughout fed-batch culture for Scramble control (gray circles), shATF6β.3 (dark purple squares), shATF6β.5 (light purple squares), and shWFS1.5 (orange triangles). On day 9, ** is for both the shATF6β.3 and shATF6β.5 pools. On day 11, the Scramble control was no longer viable, so no statistical comparison is provided. Data which were lower than our detection limit were omitted. (**f**) Overall q_P_ in pcd for each total fed-batch assay for Scramble control (gray bar), shATF6β.3 (dark purple bar), shATF6β.5 (light purple bar), and shWFS1.5 (orange bar). Data which were lower than our detection limit were omitted. Data for the shATF6β.3 pool on days 10 and 11 are shown as average ± SD *(N* = 2). Otherwise, data are shown as average ± SD (*N* = 3, **P < 0.05 Students paired t-test for two sample means).

We compared the fed-batch growth characteristics of the shATF6β.3, shATF6β.5, and shWFS1.5 pools to the Scramble control pool, all in biological triplicate (Fig. 6). The fed-batch growth characteristics of the shATF6β.3, shATF6β.5, and shWFS1.5 pools were also compared to the first fed-batch replicate of the Sham control (Supplemental Figure S9).

The mock transduction protocol did not appear to affect the IgG_1_ producer (compare the IgG_1_ producer in Fig. 1 and the Sham control in Supplemental Figure S9), however, integration of the Scramble sequence did have some effect (compare the IgG_1_ producer in Fig. 1 and the Scramble control in Fig. 6). The Scramble control and shWFS1.5 pools reached similar maximum VCDs of 5.8 ± 1.1 × 10^6^ cells/mL and 5.5 ± 0.82 × 10^6^ cells/mL, respectively, while the shATF6β.3 pool reached a maximum VCD of 4.1 ± 1.5 × 10^6^ cells/mL (Fig. 6a). While viability of all other pools dropped below 70% on day 9 of fed-batch culture, viability for the shATF6β.3 pool did not drop below 70% until day 11 (Fig. 6b). Throughout fed-batch culture, titer was lower in the shATF6β.3 pool, but this pool reached a titer of 1.7 g/L on day 11 (Fig. 6c). The Scramble control reached a similar titer of 1.6 ± 0.10 g/L on day 9; however, the maximum titer for shWFS1.5 was 1.4 ± 0.060 g/L on day 9 and was statistically lower than the Scramble control pool. Due to decreased growth, the shATF6β.3 pool reached a lower maximum IVCD compared to the Scramble control and shWFS1.5 pools (Fig. 6d). Unlike knockdown of *ATF6β*, knockdown of *WFS1* did not appear to affect growth. While there were slight differences in growth throughout fed-batch culture, both the shATF6β.3 and shATF6β.5 pools exhibited similar final titer, daily q_p_, and overall q_p_ (Fig. 6c, e, f, respectively).

Importantly, both *ATF6β* knockdown pools exhibited statistically higher daily q_P_ (day 9) and overall q_P_ compared to the Scramble control pool because of similar titer achieved with fewer cells (Fig. 6e, f, respectively). The daily q_p_ of the shATF6β.3 and shATF6β.5 pools was 62 ± 9.3 pcd and 61 ± 7.5 pcd, respectively, on day 9 compared to the daily q_P_ maximums of 47 + 2.3 and 51 ± 3.2 pcd for the shWFS1.5 and Scramble control pools on day 5, respectively (Fig. 6e). On day 11, the shATF6β.3 pool reached its maximum daily q_P_ of 76 ± 1.5 pcd. The overall q_P_ for the shATF6β.3 and shATF6β.5 pools were statistically higher at 63 ± 11 pcd and 61 ± 6.1 pcd, respectively, compared to 51 ± 7.0 pcd for the Scramble control pool and 45 + 3.9 pcd for the shWFS1.5 pool (Fig. 6f). Unlike *ATF6β* knockdown, knockdown of *WFS1* did not improve either daily or overall specific productivity during fed-batch.

### Expression of WFS1 is independent of ATF6β

**Figure 7:**
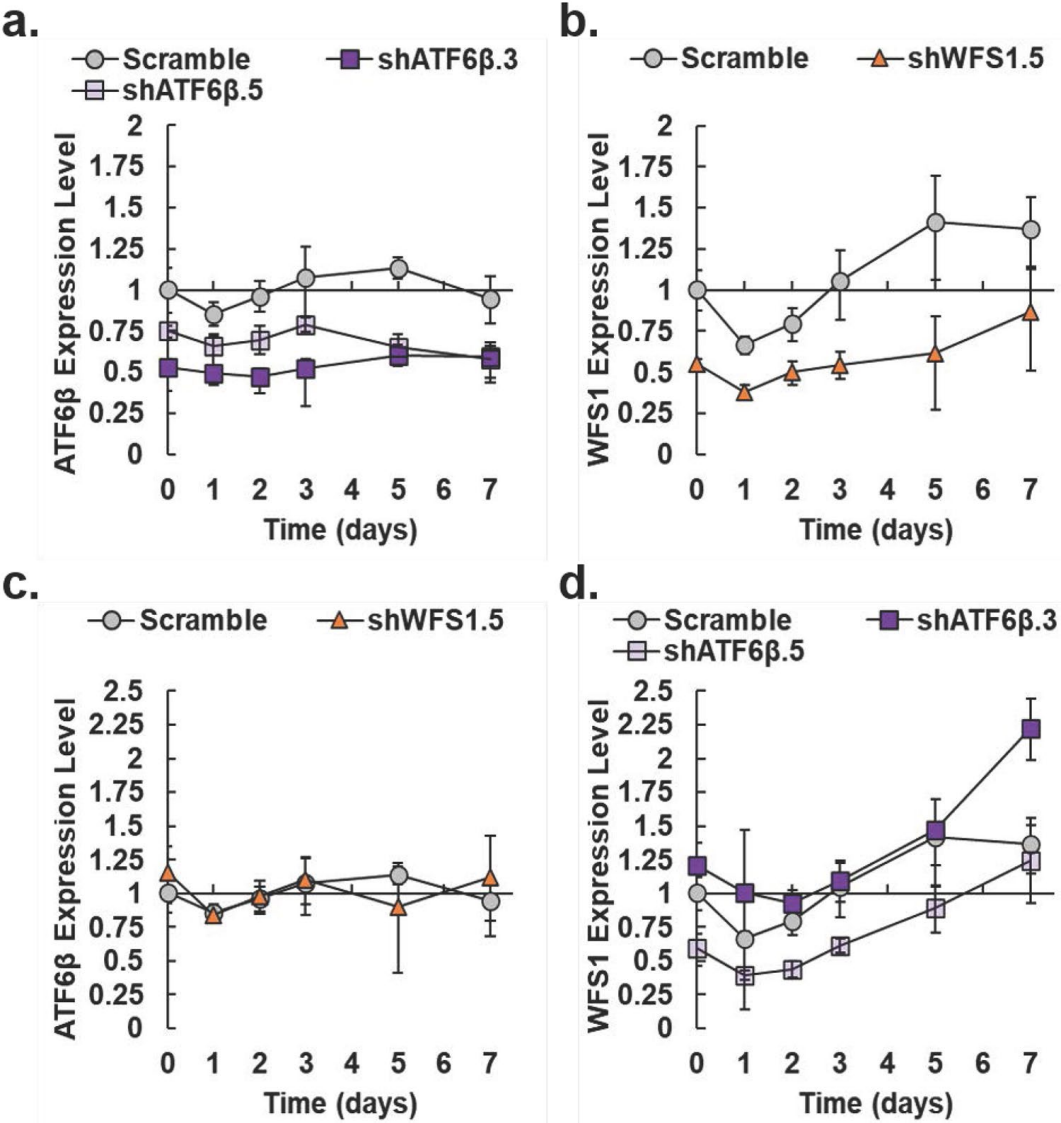
Expression levels of *ATF6β* and *WFS1* in IgG_1_-producing knockdown pools throughout fed-batch. (**a**) Expression of *ATF6β* relative to the scramble control (gray circles) for shATF6β.5 (light purple squares) and shATF6β.3 (dark purple squares). (**b**) Expression of *WFS1* relative to the scramble control (gray circles) for shWFS1.5 (orange triangles). (**c**) Relative expression of *ATF6β* in the scramble control (gray circles) and shWFS1.5 (orange triangles). (**d**) Relative expression of *WFS1* in the Scramble control (gray circles), shATF6β.5 (light purple squares), and shATF6β.3 (dark purple squares). Calculations are relative to day 0 levels for the scramble control pool. The gene β-actin was used as the reference gene. After propagating standard deviation for ∆∆Ct values, error for relative expression levels was calculated as detailed previously (*N* = 3*)*^44^. Paired Student’s *t*-test results for Fig.  7 are provided in Supplemental Table S3.

In our downstream analyses, we confirmed knockdown of *ATF6β* and *WFS1* was maintained throughout fed-batch relative to the Scramble pool (Fig. 7a, b, respectively). Knockdown of *ATF6β* was maintained throughout fed-batch in both the shATF6β.3 and shATF6β.5 pools at an average 39.9 and 35.1% on day 5 relative to the Scramble pool, respectively (Fig. 7a). Likewise, the shWFS1.5 pool maintained knockdown of *WFS1* at an average of 38.0% on day 5 relative to the Scramble control pool (Fig. 7b). In pancreatic β cells, *WFS1* is a downstream target mRNA of ATF6β^30^. As expected, knockdown of *WFS1* had no effect on *ATF6β* expression in the shWFS1.5 pool throughout fed-batch (Fig. 7c). Interestingly, expression of *WFS1* was independent of the magnitude of *ATF6β* knockdown, perhaps due to separately selected pools (i.e. different biological populations) (Fig. 7d). The shATF6β.3 pool did not exhibit a decrease in *WFS1* expression, and, in fact, exhibited increased expression of *WFS1* compared to the Scramble pool with a maximum of 2.2-fold on day 7. Conversely, expression of *WFS1* was downregulated in the shATF6β.5 pool throughout fed-batch compared to both the Scramble control and shATF6β.3 pool.

### Knockdown of ATF6β delays UPR activation without decreasing product mRNA expressions

**Figure 8:**
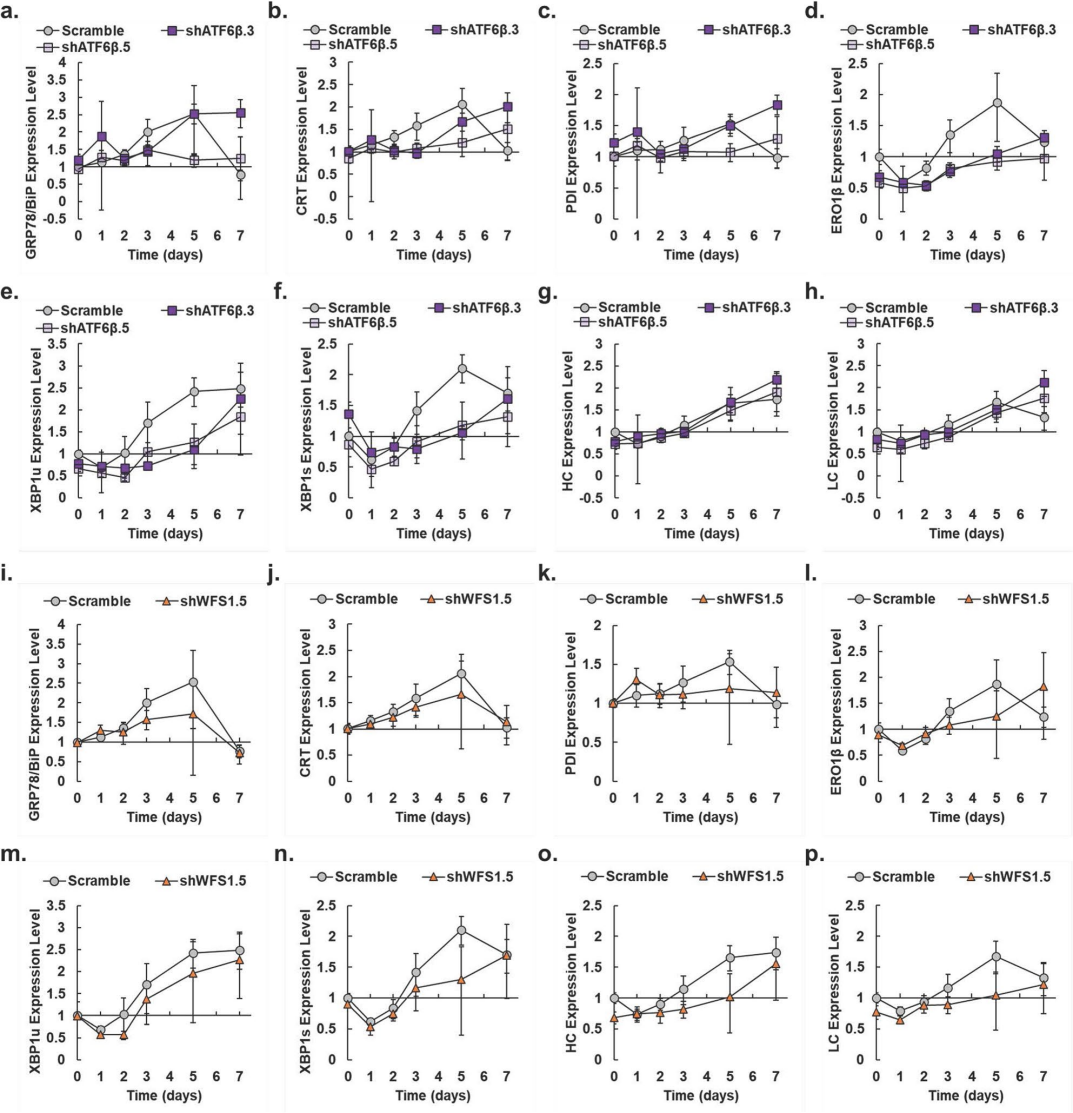
Expression level of UPR biomarkers and product mRNAs in pools as measured by qPCR. Expression levels of UPR target genes for ATF6β knockdown pools (**a**) *GRP78/BiP* (**b**) *CRT* (**c**) *PDI* (**d**) *ERO1β* (**e**) *XBP1u* (**f**) *XBP1s* (**g**) *IgG*_*1*_* Heavy Chain* (**h**) *IgG*_*1*_* Light Chain* vs day of fed-batch culture for each of the pools (Scramble, gray circles; shATF6β.3, dark purple squares; shATF6β.5, light purple squares). Expression levels of UPR target genes for the WFS1 knockdown pool (**i**) *GRP78/BiP* (**j**) *CRT* (**k**) *PDI* (**l**) *ERO1β* (**m**) *XBP1u* (**n**) *XBP1s* (**o**) *IgG*_*1*_* Heavy Chain* (**p**) *IgG*_*1*_* Light Chain* vs day of fed-batch culture for each of the pools (Scramble, gray circles; shWFS1.5, orange triangles). Calculations are relative to day 0 levels for the Scramble control pool. The β-actin gene was used as the reference gene. After propagating standard deviation for ∆∆Ct values, error for relative expression levels was calculated as detailed previously (*N* = 3)^44^. Paired Student’s *t*-test results for Fig.  8 are provided in Supplemental Table S3.

If either ATF6β or WFS1 exhibits an antagonistic relationship with ATF6α, then knockdown of either *ATF6β* or *WFS1* should boost the ATF6 pathway of the UPR and result in increased expression of downstream target mRNAs of ATF6α. We investigated the expression levels of known ATF6α target mRNAs and the product mRNAs in the knockdown pools (Fig. 8 and Supplemental Table S3). The ∆∆Ct method was used to calculate the expression level of target mRNAs relative to the reference gene β-actin and normalized to the Scramble control pool on day 0.

Knockdown of *ATF6β* increased expression of important chaperones later in fed-batch culture. Expressions of *GRP78/BiP*, *CRT*, and *PDI* in the shATF6β.3 pool reached maximums of 2.6, 2.0, and 1.8-fold on day 7, respectively, while the shATF6β.5 pool exhibited maximums of 1.5, 1.5, and 1.3-fold, respectively (Fig. 8a–c, respectively). Otherwise, the shATF6β.3 and shATF6β.5 pools exhibited similar expression profiles for other UPR biomarkers and the product mRNAs (Fig. 8d–h). In contrast to the *ATF6β* knockdown pools, the shWFS1.5 pool exhibited the lowest expression levels of *GRP78/BiP*, *CRT*, and *PDI* on day 7 (Fig. 8i–k, respectively). With respect to expression of important chaperones, these data support ATF6β, but not WFS1, as an ATF6α antagonist.

Although knockdown of *ATF6β* was expected to increase expression of *ERO1β*, *XBP1u*, and *XBP1s*, the *ATF6β* knockdown pools exhibited lower expression levels of these mRNAs than the shWFS1.5 pool (compare Fig. 8d/l, e/m, f/n), perhaps because of reduced ER stress early in fed-batch culture. The shATF6β.3 pool achieved maximum expressions of *IgG*_*1*_* heavy chain (HC)* and *IgG*_*1*_* light chain (LC)* of 2.2 and 2.1-fold, respectively, also on day 7 (Fig. 8g, h, respectively), while knockdown of *WFS1* lowered expression of these mRNAs (Fig. 8o, p, respectively), potentially causing reduced titer in the shWFS1.5 pool. The maximum expressions of chaperones and product mRNAs on day 7 in the shATF6β.3 pool shows activation of the UPR is delayed in this pool, and inhibiting the ATF6α/ATF6β antagonistic relationship helps increase overall productivity.

## Discussion

Transcriptomic analysis of a high productivity IgG_1_ producer relative to its non-producing CHO-K1 GS KO-derived host showed the UPR was activated early in the IgG_1_ producer as indicated by transient upregulation of *GRP78/BiP*. Many transcripts involved in protein processing exhibited the same, or similar, transient expression profile as *GRP78/BiP* including *CRT, PDIA3, PDIA4, PDIA6, ERO1β, HYOU1*, *CAT*, *SOD2*, and *PRDX1*. All these mRNAs are known targets of the individual or combined action of ATF6α and XBP1s^25,28,58–62^ and their collective expression suggests activation of underlying molecular phenomena: glycoprotein folding, ERAD, and oxidative stress^49,63–78^. This is significant since IgG_1_ is a glycoprotein with 14 disulfide bonds^79^. Overexpression of each of these downstream mRNAs would be time-consuming and laborious with minimal guarantee of success for improving CHO cell performance. Alternatively, targeting upstream regulators for a specific branch of the UPR, which would lead to upregulation of multiple downstream mRNAs, is a more comprehensive and strategic approach. Analysis by qPCR revealed transient upregulation of *XBP1u*, a known target of ATF6α^25,28,58–61^. Thus, engineering efforts focused on controlling the ATF6 arm of the UPR.

The transcriptomic analysis also indicated specific regulation of the ATF6 pathway in the IgG_1_ producer. The mRNA *ATF6β* was downregulated in the IgG_1_ producer, compared to the host cell line, and when compared to day 0, both the host and IgG_1_ producer showed upregulation of *WFS1* on days 3 and 5 of fed-batch culture. After identifying *ATF6β* as a target gene of microRNA miR-1287, knockdown of *ATF6β* by nucleofection increased fed-batch VCD and increased titer of a DG44-IgG_1_ CHO cell line by 1.36-fold^20^. Despite *WFS1* being identified in multiple CHO cell ER stress-related studies, there is no validation of its role in CHO cell UPR regulation^33,66,80,81^. The proteins WFS1 and ATF6β are both considered negative regulators of the ATF6 arm of the UPR with one pancreatic β cell study reporting *WFS1* as a target mRNA of ATF6β^20–22,29–32^. In fact, the gene *WFS1* was previously found upregulated in a highly proteolytic, low productivity CHO cell phenotype^33^. Collectively, these insights suggest knockdown of *WFS1* should have a similar effect as that of *ATF6β* knockdown.

We expected knockdown of *ATF6β* and *WFS1* in a CHO-K1 GS KO-derived IgG_1_-producing cell line would increase VCD and titer. In contrast to the study by Pieper et al.^20^, stable *ATF6β* knockdown in our fed-batch study showed decreases in VCD and IVCD, no significant changes in IgG_1_ titer, and, thus, increases in cell specific daily (day 9 and 11) and overall productivity. Pieper et al. observed increased specific productivity four days post transient nucleofection, but the group did not report improvements in either cell specific daily or overall productivity during fed-batch. The growth and production characteristics of the *ATF6β* knockdown pools in our study are consistent with *ATF6α* and *XBP1s* overexpression studies in CHO cells^12,47,82–84^. Although *WFS1* is a novel target in CHO cells which is also considered a negative regulator of ATF6α, knockdown of *WFS1* did not have the same effects as *ATF6β* knockdown in the IgG_1_ producer used in this study, and, in fact, decreased titer, UPR activation, and product mRNA expressions. Clonal variations, tissue types, and/or product specifics can lead to different outcomes when manipulating the UPR^12,85,86^. Different products may require different levels of UPR activation as a result of varying metabolic burden (i.e., easy-to-express versus difficult-to-express). For example, both *ATF6β* and *WFS1* were reported as upregulated in erythropoietin (EPO)-producing HEK293 cells, and *WFS1* is upregulated during insulin secretion in pancreatic β-cells^31,32,87^. Further investigation is required to better understand the benefits, or lack thereof, of *ATF6β* and *WFS1* knockdown on a product-specific basis. The effects of these engineering targets on product quality, which was not measured in this study, should also be investigated in future work.

Surprisingly, knockdown of *ATF6β* did not lower expression of *WFS1* as expected based on the results of Odisho et al.^30^, but rather, expression of *WFS1* was increased in the shATF6β.3 pool. Increased expression of *WFS1* with *ATF6β* knockdown is consistent with the effect of *ATF6β* knockout in mouse neuronal cells^88^, but it is unclear if this is due to a biological difference, product difference, or cell-line specific difference in our study. We also observed decreased expression levels of *XBP1s* in the *ATF6β* knockdown pools. Reduced *XBP1s* expression with *ATF6β* knockdown is consistent with other studies from the literature, albeit not in CHO cells, and suggests the ER load was mitigated without activating the IRE1 pathway^89^. Together, these data suggest alternative regulation mechanisms for transcription of *WFS1*. Multiple studies using a variety of cell types have reported *WFS1* as a target mRNA of either ATF6α, ATF6β, or XBP1s^20,21,29–32,66,90^. Perhaps, other transcription factors are also responsible for regulating *WFS1* in CHO cells given UPR transcription factors operate interdependently by forming homo- or heterodimers^28,58,59,61,76^. As such, expression of *WFS1*, like that of many UPR target mRNAs, may depend on an optimized ratio of available UPR transcription factors^76,89^. Also, it would not be surprising if ATF6α could promote transcription of *WFS1*, although no ER stress-response element (ERSE)-like sequence was found in the human *WFS1* promoter^91^. Multiple studies have also reported overlapping roles of ATF6α and ATF6β in which either isoform can promote transcription of target mRNAs, albeit with varying activator strengths^21,22,28,65,89,92,93^. The regulatory mechanisms between ATF6β, WFS1, and ATF6α require further investigation in CHO cells.

By day 7 of fed-batch, both *ATF6β* knockdown pools exhibited higher fold changes in expression levels of *GRP78/BiP*, *CRT*, and *PDI* in contrast to the Scramble and shWFS1.5 pools, with the shATF6β.3 pool exhibiting maximum fold changes of these chaperones. These results suggest knockdown of *ATF6β* increases UPR activation later in fed-batch culture. Our data showing *WFS1* knockdown did not increase ATF6α downstream chaperone targets (*GRP78/BiP*, *CRT*, *PDI*) later during fed-batch do not support the ATF6α antagonistic relationship previously observed in pancreatic β-cells^30,31^. In fact, upregulation of *WFS1* and expression increases of *GRP78/BiP*, *CRT*, and *PDI* were simultaneous in the shATF6β.3 pool. The proteins GRP78/BiP, CRT, and PDI participate in calcium-binding chaperone activity^64,67,68^, and in other cell types, the protein WFS1 is responsible for maintaining ER calcium homeostasis, dysregulation of which is a known cause of ER stress^64,94^. Therefore, in future studies, overexpression of *WFS1* may be a useful strategy for improving expression of needed chaperones. Future studies should also elaborate on the role of WFS1 in CHO cell ER calcium homeostasis. Unlike knockdown of *ATF6β*, knockdown of *WFS1* decreased expression levels of the product mRNAs which, in turn, could have caused reduced titer, UPR activation, and protein levels of needed chaperones and foldases. This conclusion would be better supported through translational measurements which were not conducted for this study, but should be a focus of future work.

Prolonged viability observed with the shATF6β.3 pool coincides with studies of pancreatic β-cells in which the gene *WFS1* is considered a pro-survival marker^30,31,95^, and decreased growth could provide advantages in CHO cell culture by preventing nutrient deprivation^96^. In our study, an increase in cell size and decreased glucose requirements were observed in the shATF6β.3 pool. Because *ATF6β* knockdown resulted in decreased VCD, it is expected *ATF6β* knockdown would reduce VCD for other, higher biomass cell lines, and future work should investigate these engineering targets with cell lines which achieve higher biomass. Additionally, inducible downregulation/upregulation of *ATF6β* and *WFS1* should be investigated as methods for extending CHO cell productivity by improving UPR activation at peak VCD*.*

## Conclusions

Transient upregulation of mRNAs related to glycoprotein and oxidative folding suggests IgG_1_ production is reliant on ATF6α activity. This is further bolstered by specific regulation of the ATF6 pathway in the IgG_1_ producer, and the expression profiles of *ATF6β* and *WFS1*. For the cell line used in this study, knockdown of *ATF6β* decreased growth and delayed UPR activation during fed-batch production of IgG_1_ without decreasing titer. With *ATF6β* knockdown, specific daily and overall productivity increased, but at the cost of volumetric productivity In contrast, knockdown of *WFS1* did not adversely affect growth but decreased titer and product mRNA expressions. In our study, upregulation of *WFS1* in one *ATF6β* knockdown pool also coincided with decreased growth and upregulation of ER chaperone mRNAs suggesting overexpression of *WFS1* may be a successful strategy for managing the CHO cell UPR during production. Overexpression of UPR transcription factors does not always yield improved cell line performance, and there is a need to use new strategies for tailoring UPR activation, such as engineering upstream regulators of the UPR. This study demonstrates usefulness of transcriptomic analyses of CHO cell production over time to identify rational engineering targets, such as *ATF6β* and *WFS1*. Furthermore, this approach can be applied to CHO cell lines producing different therapeutics. Our results demonstrate the importance of regulating the ATF6 pathway as a means of managing CHO cell growth and UPR activation during fed-batch production of IgG_1_.

### Supplementary Information

Supplementary Information.

## Data Availability

Sequencing data has been deposited in the National Center for Biotechnology Information’s Gene Expression Omnibus database, URL accession number GSE217637: https://www.ncbi.nlm.nih.gov/geo/query/acc.cgi?acc=GSE217637. All other datasets are available from the corresponding author upon request.
