# Supplementary material for: RNASeq highlights ATF6 pathway regulators for CHO cell engineering with different impacts of ATF6β and WFS1 knockdown on fed-batch production of IgG1
Source: Sci Rep. 2024 Jun 19;14:14141. doi: 10.1038/s41598-024-64767-1 (PMC11187196; doi:10.1038/s41598-024-64767-1)
Supplement: Supplementary file 1 — Supplementary Information. [file 41598_2024_64767_MOESM1_ESM.docx]

**Supplemental Tables**

**Supplemental Table S1: Primer Sequences for UPR Biomarkers, ATF6α Target mRNAs, and Product mRNAs used in qPCR Analyses**

| **Target** | **Primers** | **Reference** |
| --- | --- | --- |
| GRP78/BiP | F 5' to 3' TGGGTACATTTGATCTGACTGGA  R 5' to 3' CTCAAAGGTGACTTCAATCTGGG | 15 |
| EDEM | F 5' to 3' CTACCTGCGAAGAGGCCG  R 5' to 3' GTTCATGAGCTGCCCACTGA |  |
| GRP94 | F 5' to 3' AAGAATGAAGGAGAAGCAGGACAAAA  R 5' to 3' CAAATGGAGAAGATTCGGC |  |
| PDI/P4HB | F 5' to 3' GGACAAACTGGGAGAGACATACA  R 5' to 3' ACTTTGACGGCTTCCACCTC | in-house design |
| XBP1* | Unspliced XBP1 (XBP1u):  F 5' to 3' GCAGCACTCAGACTACGTGCA  Spliced XBP1(XBP1s):  F 5' to 3' TGCTGAGTCCGAATCAGGTGCAG  R 5' to 3' GTCAGAATCCATGGGAAGATGTTCTGG | 15; redesigned for 60˚C |
| ATF4 | F 5' to 3' ATTCTCCGGGACAGACTGGA  R 5' to 3' TGGCCAATTGGGTTCACTGT | 97 |
| CHOP | F 5' to 3' CGAACCAGGAAACGGAAACAGA  R 5' to 3' TCTCCTTCATGCGCTGCTTC | 15 |
| ATF6β | F 5’ to 3’ GCGGGAAGACACCTTCTATGTT  R 5’ to 3’ CAGGGACATCTTGGGTCTGG | in-house design |
| WFS1 | F 5’ to 3’ TTTGTGTGTGTGTTGGATTTG  R 5’ to 3’ CAGCCACTGTCAAGGATTAG | in-house design |
| CRT | F 5’ to 3 ACCTGCCGTCTATTTCAAAGAGC  R 5’ to 3’ TCCCCGTAGAATTTGCCAGAACT | 98 |
| ERO1β | F 5’ to 3’ TTGGTGAAGGTGGGTAAGTG  R 5’ to 3’ AGGAGAGGAACAAAGAGAGAAG | in-house design |
| IgG Heavy Chain (HC) | F 5’ to 3’ GCTGAATGGCAAGGAGTACAAG  R 5’ to 3’ CCCTTTGGCTTTGGAGATGG | in-house design |
| IgG Light Chain (LC) | F 5’ to 3’ GATCGCTTCTCTGGCTCCAA  R 5’ to 3’ AGCAGCAGTAATAATCAGCCTC | in-house design |
| β-actin | F 5' to 3' AAAGACCTCTATGCCAACACA  R 5' to 3' GCAGTGATCTCCTTCTGCATC | in-house design |

* Underlined bases represent mismatches introduced to reduce background between primers binding to both unspliced and spliced forms of XBP1. Base adenine (A) was originally cytosine (C) and base thymine (T) was originally guanine (G), respectively.

**Supplemental Table S2: Unpaired Student’s t-test Results for qPCR Analysis of UPR Activation in Fed-batch Samples of the Host Cell Line and IgG_1_ Producer**

**
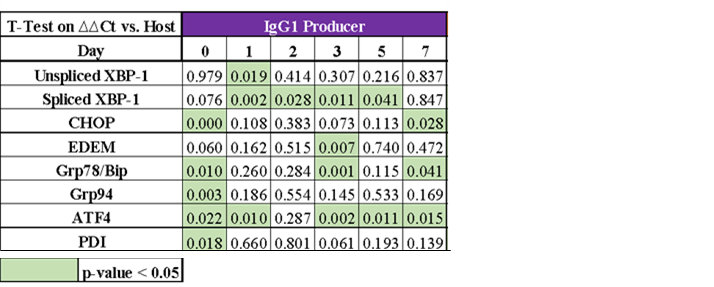
**

**Supplemental Table S3: Paired Student’s t-test Results for qPCR Analysis of ATF6β and WFS1mRNAs, ATF6α Target mRNAs, and Product mRNAs in Chosen Knockdown Pools of the IgG_1_ Producer**

**Supplemental Figures**

**
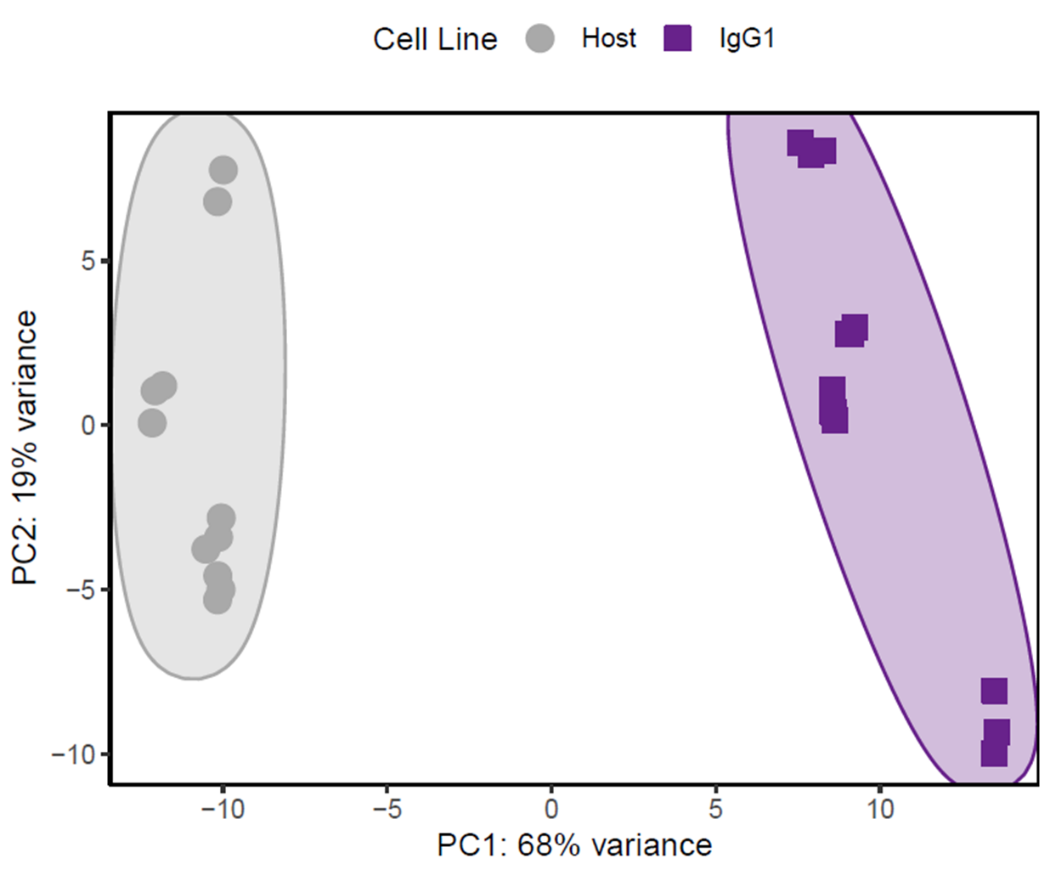
**

**Supplemental Figure S1: Principal Component Analysis (PCA) of RNASeq Dataset** Each dot represents a sample replicate from fed-batch culture for the host cell line (gray circles) or IgG_1_ producer (purple squares).

**
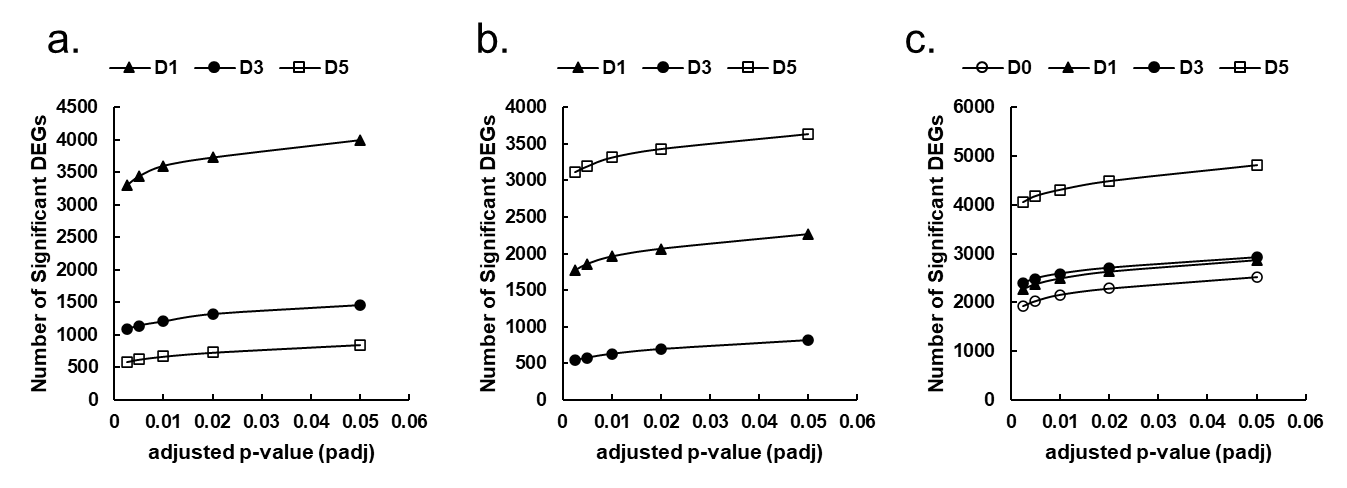
**

**Supplemental Figure S2: Number of Significant Differentially Expressed Genes (DEGs) Versus Varying adjusted p-value (padj) a.** Number of DEGs for the host cell line versus the host cell line day 0 for day 1 (D1, filled triangle), day 3 (D3, filled circle), day 5 (D5, open square). **b.** Number of DEGs for the IgG_1_ producer versus the IgG_1_ producer day 0 for day 1 (D1, filled triangle), day 3 (D3, filled circle), day 5 (D5, open square). **c.** Number of DEGs for the IgG_1_ producer versus the host cell line for day 0 (D0, open circle), day 1 (D1, filled triangle), day 3 (D3, filled circle), day 5 (D5, open square).


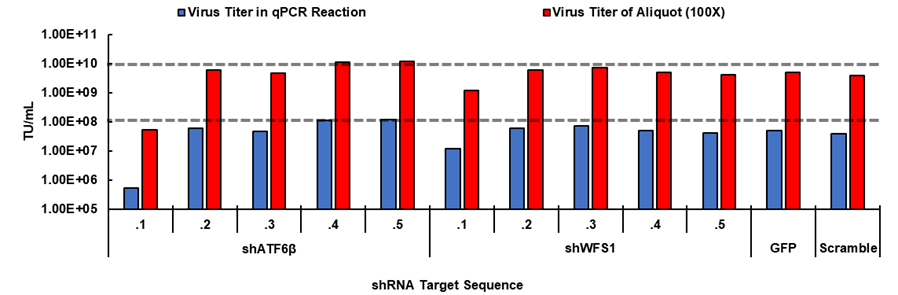


**Supplemental Figure S3: Titer of Lentivirus in TU/mL Measured via qPCR using Applied Biological Materials qPCR Lentivirus Titration Kit (Cat. No. LV900)** Aliquots of lentivirus (red bars) were run at 100X dilution in qPCR reactions (blue bars). Five sequences were used to target *ATF6β* or *WFS1* as well as non-targeting controls expressing green fluorescent protein (GFP) and Scramble sequence. Dashed lines represent highest titer achieved for aliquots (top line) and qPCR reactions (bottom line).

**
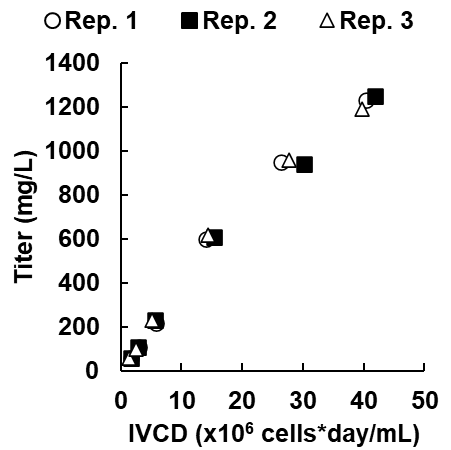
**

**Supplemental Figure S4: Titer of IgG_1_ (mg/L) versus IVCD (x10^6^ cells*day/mL) in the IgG_1_ Producer.** Data points shown for Replicate 1 (white circles), Replicate 2 (black squares), and Replicate 3 (white triangles) fed-batch cultures of the IgG_1_-producing cell line.


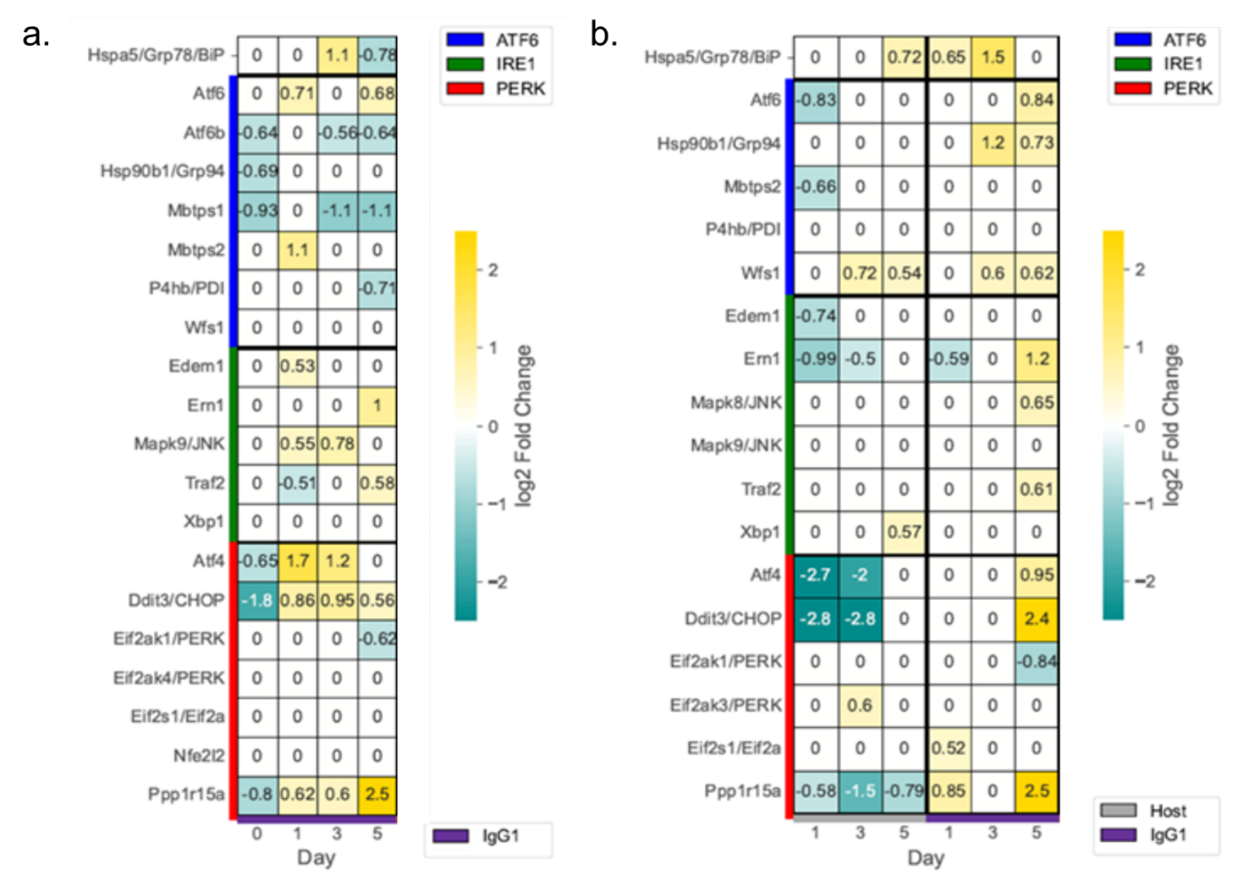


**Supplemental Figure S5: Quantitative Heatmap of Differentially Expressed UPR mRNAs of Host and IgG_1_-producing Cell Lines Versus a. Host Cell Line and b. Day 0 Controls** Differentially expressed transcripts have an absolute value of the log2FC greater than 0.5 and an adjusted p-value less than 0.01 *(N = 3)*. Fold change equals 2^log2FC^, where log2FC is an absolute value. Transcripts which are upregulated are shown in gold, and transcripts which are downregulated are shown in cyan. Left legends shows transcripts as organized by UPR pathways ATF6 (blue), IRE1 (green), and PERK (red). **a.** Values of log2FC are shown in individual squares representing a specific day in fed-batch culture versus the host cell line for the IgG_1_-producing cell line. The bottom legend shows samples for the IgG_1_ producer (purple). **b.** Values of log2FC are shown in individual squares representing a specific day in fed-batch culture versus day 0 for the respective cell line. The bottom legend shows samples for the host cell line (gray) and the IgG_1_ producer (purple).


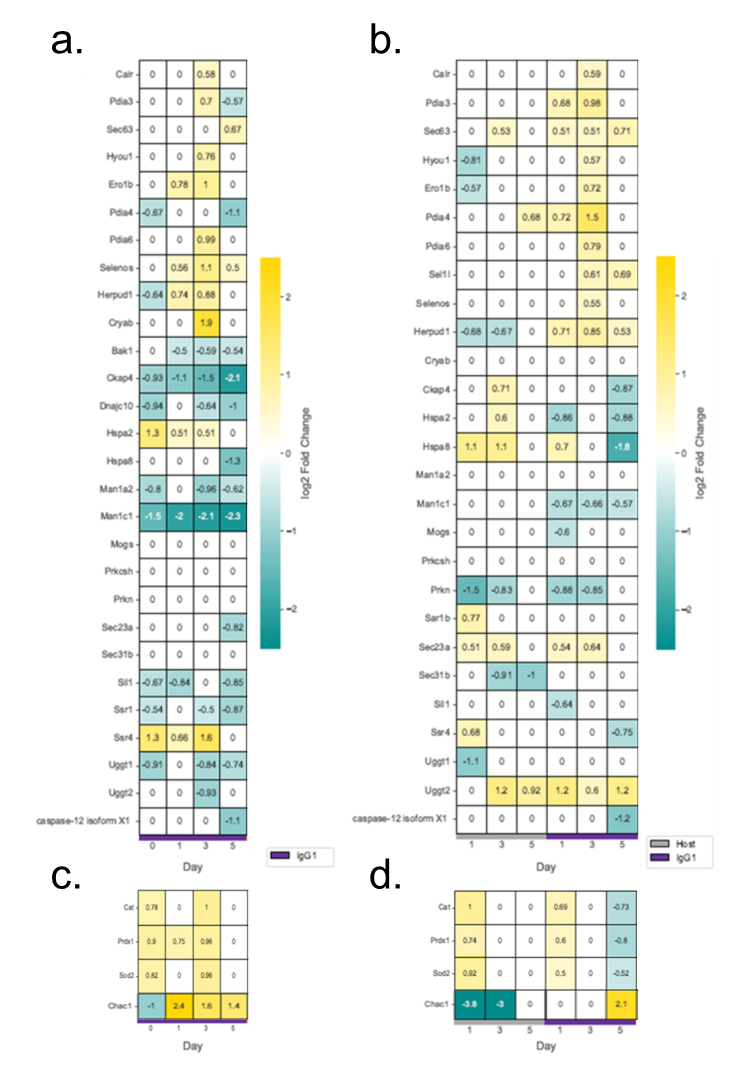


**Supplemental Figure S6: Quantitative Heatmap of Differentially Expressed Protein Processing and Oxidative Folding mRNAs of All Cell Lines Versus a./c. Host Cell Line and b./d. Day 0 Controls** Differentially expressed transcripts have an absolute value of the log2FC greater than 0.5 and an adjusted p-value less than 0.01 *(N = 3)*. Fold change equals 2^log2FC^, where log2FC is an absolute value. Transcripts which are upregulated are shown in gold, and transcripts which are downregulated are shown in cyan. **a.** Values of Protein Processing mRNAs where log2FC are shown in individual squares representing a specific day in fed-batch culture versus the host cell line for the IgG_1_-producing cell line. The bottom legend shows samples for the IgG_1_ producer (purple). **b**. Values of Protein Processing mRNAs where log2FC are shown in individual squares representing a specific day in fed-batch culture versus day 0 for the respective cell line. The bottom legend shows samples for the host cell line (gray) and the IgG_1_ producer (purple). **c.** Values of Oxidative Folding mRNAs where log2FC are shown in individual squares representing a specific day in fed-batch culture versus the host cell line for the IgG_1_-producing cell line. Scale and legend are the same as in Figure a. **d.** Values of Oxidative Folding mRNAs where log2FC are shown in individual squares representing a specific day in fed-batch culture versus day 0 for the respective cell line. Scale and legend are the same as in Figure b.


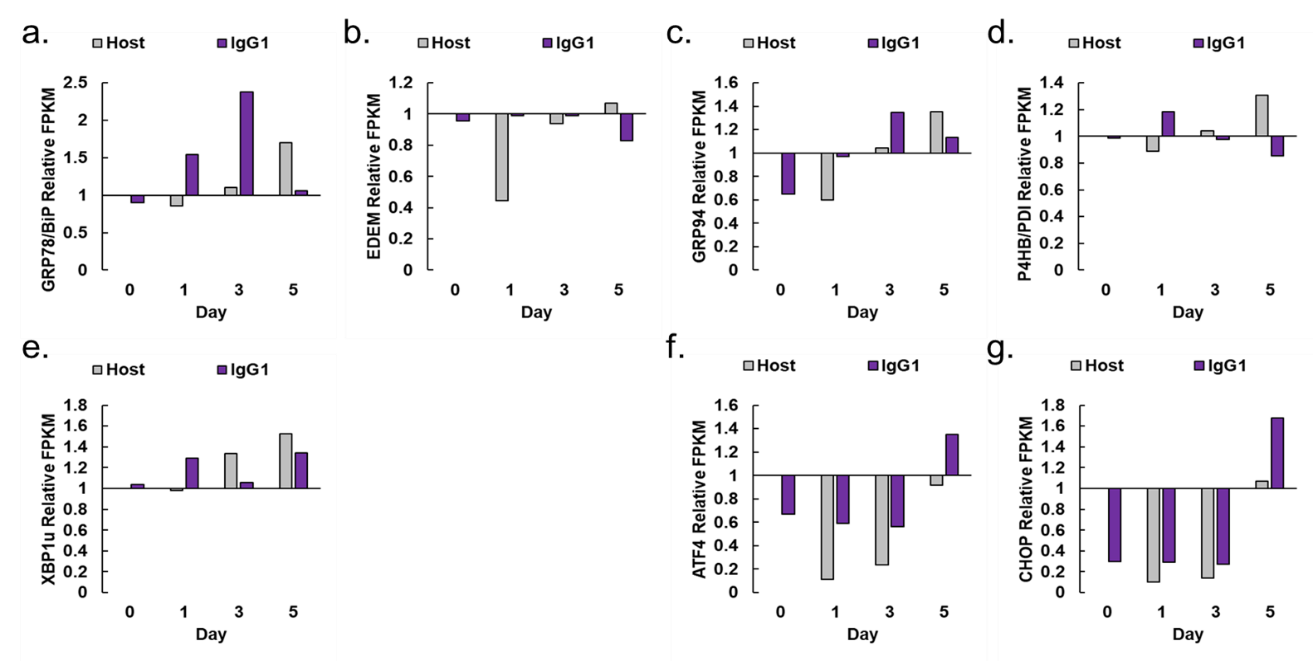


**Supplemental Figure S7: Fragments Per Kilobasepair Million (FPKM) of UPR Markers Relative to β-actin and Host, Day 0 Values as Measured by High Throughput RNA Sequencing (RNASeq)** FPKM of UPR target genes **a.** *GRP78/BiP* **b.** *EDEM* **c.** *GRP94* **d.** *PDI* **e.** *XBP1u* **f.** *ATF4* **g.** *CHOP* vs. day of fed-batch culture for each of the cell lines (IgG_1_ producer, purple squares; host, gray circles). Calculations are relative to day 0 levels for the host cell line. The β-actin gene was used as a housekeeping gene.


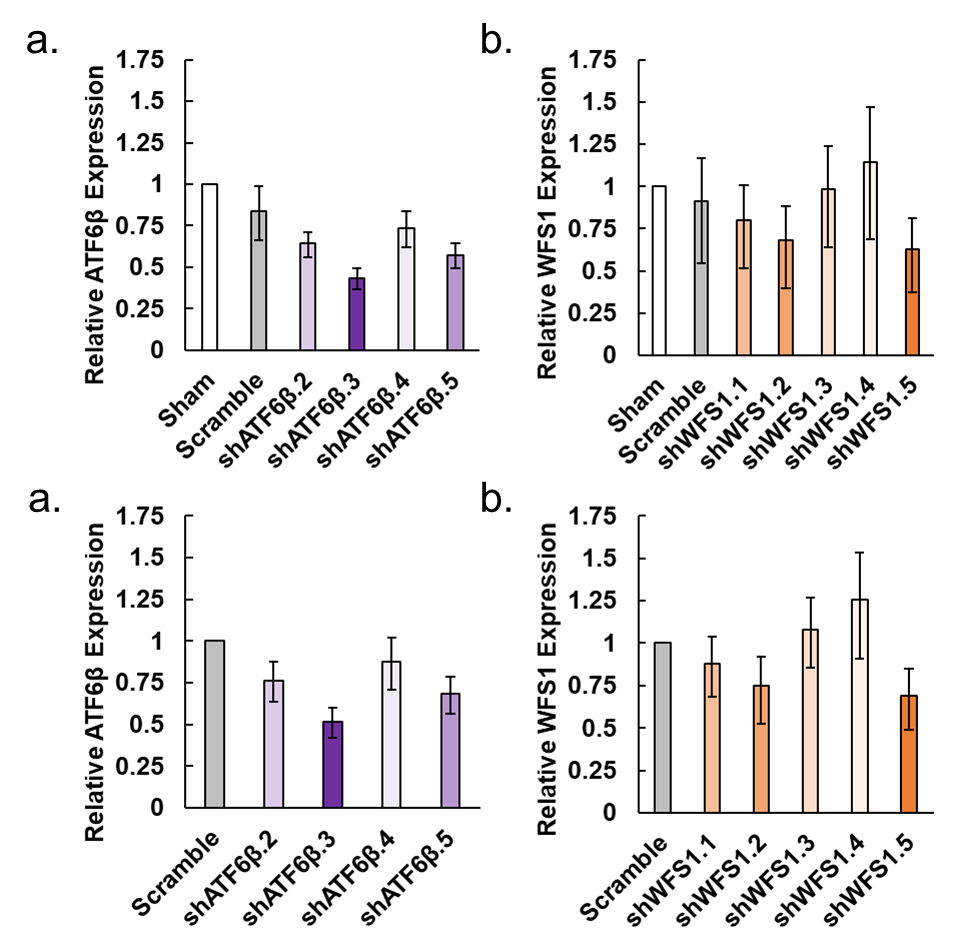


**Supplemental Figure S8: Relative Expression of *ATF6β* and *WFS1* in Selected IgG_1_-Producing Pools on Day 5 of Fed-batch a.** Expression of ATF6β relative to the Sham control (white bar) for the Scramble control (gray bar) and shATF6β knockdown pools (purple bars). **b.** Expression of WFS1 relative to the Sham control (white bar) for the Scramble control (gray bar) and shWFS1 knockdown pools (orange bars). All calculations are relative to day 5 levels. The β-actin gene was used as a housekeeping gene. After propagating standard deviation for ∆∆Ct values, error for relative expression levels was calculated as detailed previously *(N = 3)*^43^.


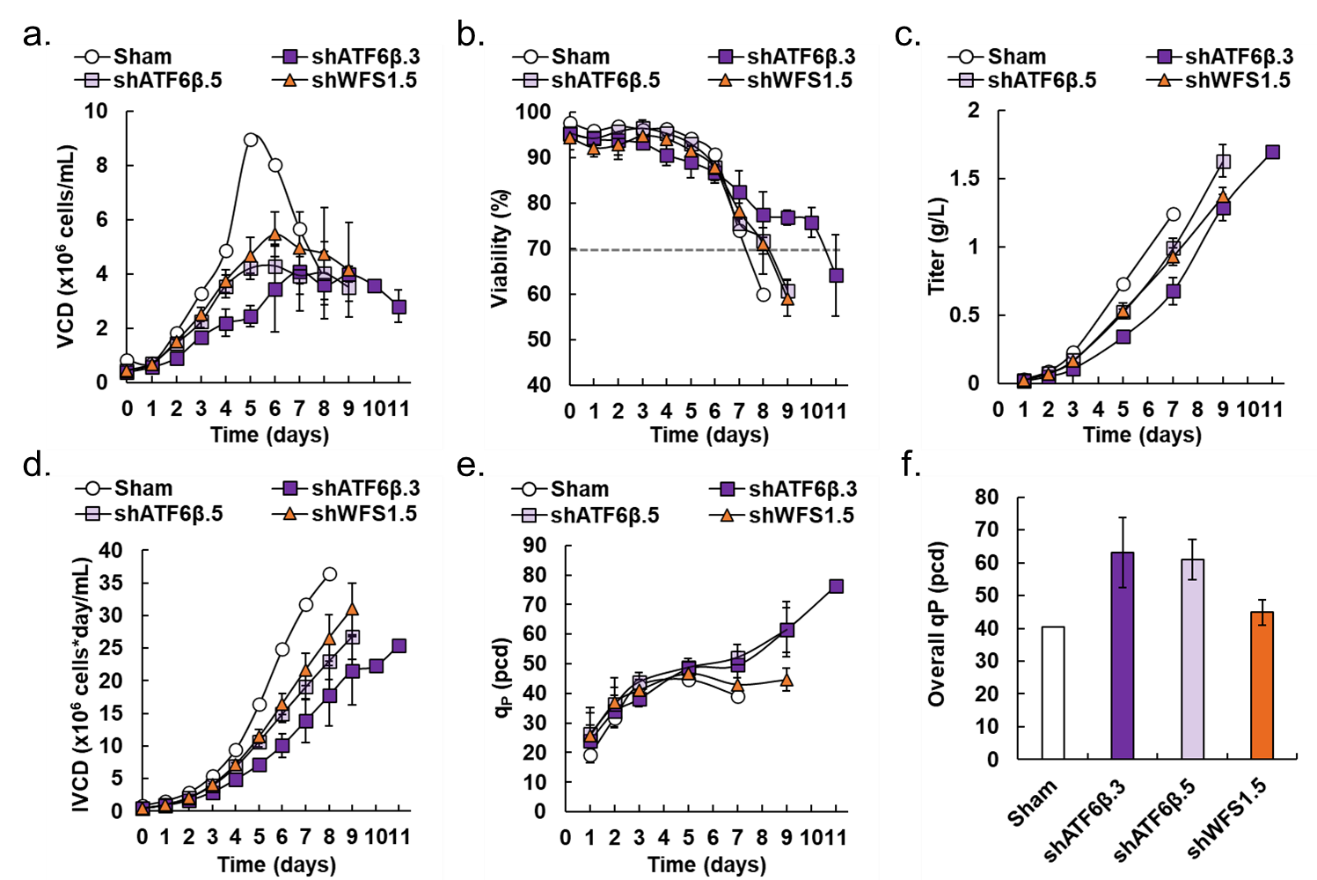


**Supplemental Figure S9: Growth Characteristics of *ATF6β* Knockdown Pools Compared to Scramble Control Pool a.** Viable Cell Density (VCD) in x10^6^ cells/mL for Sham (white circles), shATF6β.3 (dark purple squares), shATF6β.5 (light purple squares), and shWFS1.5 (orange triangles). **b.** Viability as a percentage for Sham (white circles), shATF6β.3 (dark purple squares), shATF6β.5 (light purple squares), and shWFS1.5 (orange triangles). Dashed line represents 70% viability. **c.** Titer in g/L for Sham (white circles), shATF6β.3 (dark purple squares), shATF6β.5 (light purple squares), and shWFS1.5 (orange triangles). Data which were lower than our detection limit were omitted. **d.** Integral of VCD (IVCD) in x10^6^ cells*day/mL for Sham (white circles), shATF6β.3 (dark purple squares), shATF6β.5 (light purple squares), and shWFS1.5 (orange triangles). **e.** Specific daily productivity (q_P_) in pcd throughout fed-batch culture for Sham (white circles), shATF6β.3 (dark purple squares), shATF6β.5 (light purple squares), and shWFS1.5 (orange triangles). Data which were lower than our detection limit were omitted. **f.** Overall q_P_ in pcd for each total fed-batch assay for Sham (white bar), shATF6β.3 (dark purple bar), shATF6β.5 (light purple bar), and shWFS1.5 (orange bar). Data which were lower than our detection limit were omitted. For the Sham control, single replicate data are shown (*N=1*). Data for the shATF6β.3 pool on days 10 and 11 are shown as average + SD *(N=2)*. Otherwise, all other data are shown as average + SD (*N=3)*.
